# Centralized or decentralized perinatal surgical care for rural women: a realist review of the evidence on safety

**DOI:** 10.1186/s12913-016-1629-6

**Published:** 2016-08-13

**Authors:** Jude Kornelsen, Kevin McCartney, Kim Williams

**Affiliations:** 1 Department of Family Practice, University of British Columbia, David Strangway Building, 3rd Floor, 5950 University Blvd., Vancouver, V6T 1Z3 BC Canada; 2 Perinatal Services BC, Provincial Health Services Authority, West Tower, Suite 350, West 12th Ave., Vancouver, V5Z 3X7 BC Canada

**Keywords:** General practitioners with enhanced surgical skills, GP proceduralists, GP surgeons, Rural maternity care, Rural surgical care, Realist review

## Abstract

**Background:**

The precipitous closure of rural maternity services in British Columbia (BC), Canada, and internationally has demanded a reevaluation of how to meet the perinatal surgical needs of rural women in accordance with the Triple Aim objectives of safety, cost-effectiveness, and satisfaction of all key stakeholders. There is emerging international evidence that General Practitioners with Enhanced Surgical Skills (GPESS) are a well-positioned health service solution due to their generalist nature in low-volume settings. A realist review was undertaken to evaluate international evidence on efficacious models of perinatal surgical care. This article presents findings of the safety of such practice, one discrete part of the full realist review.

**Methods:**

This paper was derived from a larger review, which used a *realist review* methodology to guide the approach, and adhered to the RAMESES quality standard for realist reviews. Seven academic databases were searched in December 2013, using year (1990) and language (English) limiters in keeping with a rapid review approach. Mining of bibliographies in addition to consultation with international experts led to further inclusion of academic and grey literature up to March 2014.

**Results:**

Two hundred fifty-four articles were originally identified; 119 articles were removed from consideration for lack of fit, resulting in the review of 191 articles from the peer reviewed and grey literature. Of these, 53 pertained to safety and are considered herein. Evidence on the safety of GPESS was consistent in the literature cited. Clinical, case study, and qualitative evidence demonstrates that perinatal surgical care is equally safe when provided by GPESS and specialist physicians.

**Conclusion:**

Findings allow health planners to confidently build perinatal surgical services around the contribution of GPs with enhanced surgical skills and focus on educational, regulatory, and continuing professional development mechanisms to ensure their sustainability. Volume-to-outcomes associations are variable and inconclusive with regards to safety, suggesting the need for more evidence. These findings, and the attendant health services planning directions, are reassuring as they suggest the viability of local models of care where feasible.

**Electronic supplementary material:**

The online version of this article (doi:10.1186/s12913-016-1629-6) contains supplementary material, which is available to authorized users.

## Background

Across Canada and internationally, we have seen the precipitous closure of many rural health services [1–5], leading in some instances to deteriorating population health outcomes and reduced quality of care [5–8]. Currently, professional trends in General Surgery and Obstetrics have led to a reduction in their contribution to smaller rural services [9, 10]. This is occurring within a research and policy context that recognizes the benefits of services “closer to home” [11, 12] balanced with the need for fiscally responsible planning [4]. In small-volume centres, a generalist approach has been shown to be the most efficacious way of meeting the needs of the population [10, 13, 14]. In the case of cesarean section, these conditions have created a response from General Practitioners with Enhanced Surgical Skills (GPESS) training to meet the operative needs of the population in many jurisdictions. The primary care focus of their work alongside their availability for limited procedural work addresses the challenges of low surgical volume in conjunction with the primary care needs of rural communities. Although this solution has been recognized and integrated into rural health care planning in jurisdictions such as Australia, the United States, Norway, Scotland, and in more ad-hoc ways in Canada and other jurisdictions, a review of the international literature on the safety and outcomes of GPESS has not been undertaken. In 2012, the BC Ministry of Health held province-wide consultations with key stakeholders in order to establish a set of consensus-driven action items for a provincial primary maternity care agenda, known as the BC Primary Care Plan. These consultations also resulted in a series of short-term “action items.” Health care decision makers recognized that any reasoned debate about these issues demanded a rigorous review of the international literature.

In addition to the BC Primary Maternity Care Plan, perinatal planning in British Columbia (BC) has been conceptually guided by a report authored by Justice Peter Seaton in response to the Royal Commission of Health Care and Costs, which recommended “[m]edically necessary services… be provided in, or as near to, the patient’s place of residence as is consistent with quality and cost-effective health care” (P. A-6). This recommendation was made based on two features. First, the Seaton report recognizes the challenges rural residents face in accessing health care, including insufficient supply of providers, inappropriate emergency services and the cost incurred by patients forced to travel for treatment [15]. These same challenges are faced by rural residents in various international jurisdictions. Second, the Seaton report expressed the belief that a decentralized health care system would better respond to many health needs within rural and remote communities.

The fundamental challenge to providing operative backup for deliveries in rural communities internationally is lack of availability of surgical providers [16]. This has become the reality in rural British Columbia as well [17]. The solution pursued worldwide is to increase the supply of rural generalist surgeons, including training more General Practitioners with Enhanced Surgical Skills and involving more General Surgeons in the delivery of perinatal surgical services. The relatively small procedural volumes of these programs, however, are associated with important issues regarding program sustainability – which deter specialist practice – including the challenge of maintaining competence for the professional staff, lack of opportunity for intensive application of practitioners’ skills, restriction on the numbers of skilled providers that can be supported by the local service demand (leading to vacation and on-call relief problems), and programs associated with high unit costs. Despite this, research evidence has demonstrated the importance of local cesarean section in sustaining rural maternity services [18, 19]. A BC study found local access to cesarean section increased the proportion of local deliveries from less than 30 % (no local cesarean section) to greater than 75 % when operative deliveries were locally available [20, 21]. Similarly, a study in Alberta found a local retention rate of 22.1 % for women in communities without cesarean section compared to 70.1 % in communities with local operative delivery [22].

In the early 1990s, evidence began emerging which suggested that the profession of General Surgery was aging and due to inevitable retirement would not be able to sustain a strong rural presence without training new practitioners [9, 14, 23–25]. However, attracting new recruits was difficult due to the perception of lack of interest in the specialty, leading to demanding call schedules and the lack of sub-specialist support in rural environments [10]. This is despite the recommendations of the Barer-Stoddart report [26], which suggested priority be given to training generalist surgeons for practice in non-urban hospitals. The lack of General Surgeons in rural areas is not unique to Canada but also characteristic of rural Australia [27] and the United States [28–30].

The reality in British Columbia is most rural areas are not serviced by local specialist support: General Practitioners with Enhanced Surgical Skills are the primary surgical service provider [14, 31, 32], making the GPESS model synonymous with “decentralized perinatal surgical services.” For populations of 5000–15,000, surgical services are provided locally by one or more GPESS, cesarean section often being the backbone to their procedural skills repertoire. For populations of 15,000–25,000, there is usually a specialist surgeon, in some instances an obstetrician, supported by one or more GPESS (a “mixed” model). In these larger communities, the GPESS provides call relief and often covers the operative delivery program. For populations greater than 25,000, there are usually groups of specialists without any GPESS [32].

In 1995/96, the most recent published data, 1838 c-sections were performed by 200 rural GPs in Canada [33]. Rural intrapartum care was provided by 1704 rural GPs, who attended 25,602 births, 8.4 % of total births in Canada that year [33]. Three-quarters of all GPs performing c-sections were doing so west of Ontario [33], and GPs with Enhanced Surgical Skills practiced at 60 of the 72 small rural hospitals (<51 beds, <15,000 person catchment) providing surgical services in BC, Alberta, Yukon, and the Northwest Territories [34]. Forty-three of those hospitals had GPs performing c-section procedures [34].

Given this context, this review sought international literature on models of care to meet the perinatal surgical needs of rural women in order to provide a broader context to rural health planning in British Columbia. Although GPESS is synonymous with decentralized perinatal surgical care in BC, this review considered all models in jurisdictions with a comparable health services context. Due to space limitations, this paper focuses exclusively on evidence of the safety and outcomes of models reviewed.

This realist review was commissioned by Perinatal Services of British Columbia (PSBC), a provincial policy body in BC, as part of a provincial strategic planning process to establish an evidence-informed primary maternity care agenda. One of the action items resulting from the agenda was focused on resolving some of the inter-professional and regulatory tensions within the medical community regarding GPs with Enhanced Surgical Skills and their role in sustaining perinatal surgical services for rural women. As GPESS were seen as underscoring only one potential model of care, the review question was structured to be purposively open to evidence suggesting the effectiveness of a more centralized response as well (i.e., moving rural women into regional maternity care units for labor and delivery). The final research question was:

*Can we meet the perinatal surgical needs of rural women more effectively through an optimally centralized or optimally decentralized model of care?*

Commissioners felt that exploring what is known from other jurisdictions, as well as from BC and across Canada, in a systematic and comprehensive way would provide the scaffolding on which to build a framework to address conditions in British Columbia. Although the entirety of the review covered five discrete themes, the focus of this paper is on what was learned from research literature on safety and outcomes. The frequent lack of policy and service context found in academic literature is a considerable barrier to inter-jurisdictional learning [35], and so the contextual features of BC are made explicit with the intention of improving the international applicability of the findings. The findings from other themes are presented elsewhere. The full report is publically available [36].

## Methods

In health service research, traditional meta- and systematic reviews have significant limitations for stakeholders in jurisdictions outside of the review setting. Context at every level, including health system structures, health professional relationships, historical precedent, and community expectations all impact the portability of solutions from setting to setting. Given this, it was determined that an efficacious way to look at models of health care delivery and their applicability to the British Columbia context was through a *realist review* method, which brings a mandate to examine the totality of evidence on a research question with appropriate consideration for the dynamic policy and practice landscape in which that evidence was embedded. This method allows researchers to consider new questions and directions as the literature is examined [37], particularly useful when searching for models of care from other jurisdictions. It is based on an approach Wong et al. [38] call “CMO”: understanding the complex relationship of Context, Mechanism, and Outcome. In addition to being contextually located, evidence included in a realist review is broad, reflective of the variety of influencing factors involved.

The RAMESES quality standard for realist reviews guided the methodology [39] with the current study meeting an excellent standard by most criteria (i.e., feasible topic, appropriately structured question, understanding and application of realist philosophy, rigor of appraisal process).

This study emerged from a larger review initiated to address an evidence gap in best practices for meeting the perinatal surgical needs of rural women guided by the question “*Can we meet the perinatal surgical needs of rural women more effectively through an optimally centralized or optimally decentralized model of care?*” Evidence was requested by Perinatal Services of British Columbia with a particular focus on optimal levels of (de)centralization within a planning context of budget constraint.

Inclusion criteria for the search were research findings published in the English language since 1990 with at least one search term from each of three areas (see Table 1). Placing limitations on the search parameters is consistent with a rapid review approach. Rapid review methods are often considered in relation to full systematic reviews and have become common place in many health disciplines. Rapid realist methodology, however, is still emerging and is not yet well defined. The procedures of this research were held up to the scrutiny of rigorous realist review guidelines, while the limitations placed on the search, the timeframe of the full review (under 6 months), and the close involvement of an end-user are in keeping with a rapid review definition.

**Table 1:** Search Terms and Keywords. Search terms and areas for inclusion criteria of literature search

Search area	Keywords	Reasoning
Maternal / Perinatal Health	obstetric* matern* perinatal reproduct* (birth or birthing) parturi*	This review focuses on maternal and obstetric care, and so appropriate terms were furnished to limit the search to that singular area of care.
Perinatal Surgical Care	surgery surgical (cesarean or caesarean or c-section*)	We aimed for a broad surgical requirement, rather than an exhaustive list of obstetric surgeries.
Rural and Remote Health Services	(decentral* or de-central*) rural health* rural hospitals rural communit* remote health* remote communit* “hub and spoke” rural remote	The review seeks to compare models of centralized and decentralized care. Increasingly since 1990, centralization of care has been the backdrop of studies regarding decentralized models. Moreover, this review seeks to compare models of care in their ability to provide safe, high quality, cost effective perinatal surgical care to rural women specifically, and so rural health was a required search subject.

A broad and iterative approach to the search terms was particularly important due to the consolidated nature of the question (evaluations of optimally centralized or decentralized models) and the need to consider the thematic areas that would address the question (safety, outcomes, sustainability, costs, satisfaction). We searched MEDLINE, PubMed, EMBASE, CINAHL, EBM Reviews, NHS Economic Evaluation Database, and PAIS International for literature. The primary search was completed in December 2013. Grey literature was obtained from Perinatal Services of British Columbia and the SAX Institute of Australia, and the review team mined bibliographies for further academic and grey resources, through March 2014.

The further inclusion criteria were applied at the review stage of the full body articles that account for a relatively high rate (30 %; 57 of 192) of exclusion upon full article review. Articles were included only if they focused on direct discussions of maternal surgical care, including but not limited to safety of practice models, governance of care models, and sustainability of service delivery. Articles were also included on the centralization of decision making, ways of incorporating specialist care into service models, and optimal geography and/or level of service delivery. Much of the literature excluded at the full article review stage was focused on internist, general, or other non-obstetric surgery for rural patients.

Literature from low- and middle-resource settings was excluded manually due to lack of fit with BC’s health service delivery context. While BC and the world have much to learn from such health settings, there are meaningful validity problems to synthesizing across distinctly different health contexts around the world, especially when applied to a question regarding medicalized, surgical intervention. The expectation of a realist review includes answering which interventions work for whom under what circumstances, such that material and cultural differences in health service settings challenge the appropriateness of a single review from all jurisdictions. Search parameters did include all settings, however. Consequently, literature was included from international contexts deemed relevant to the context in BC: Scotland, the United Kingdom, Norway, Finland, Sweden, Holland, Germany, New Zealand, the United States, Australia, and the rest of Canada.

The lead reviewer reviewed articles selected for inclusion and extracted appropriate data that was then reviewed by the lead author. A sub-set of articles (8) was reviewed by additional reviewers and compared for consistency of extracted data. There was a high degree of consistency between reviewers.

Although the types of evidence found in the search were varied, the majority of studies were descriptive in nature. Case studies, service reviews and chart audits including retrospective chart reviews were common, often using population level data at the national or regional level. Additionally, several studies used chart reviews to compare outcomes from specialist obstetric surgical care to generalist care. Program or intervention research was a smaller portion of the research than expected, perhaps because of our focus on models of care rather than smaller units of health service delivery. Still, a handful of articles detailed trials of new models of care, including specialist outreach and telehealth. Finally, editorials and grey literature reports that were found with the help of policy and service programming experts in both Canada and Australia were also included. There were no existing systematic reviews or randomized controlled trials in the body of evidence. When considered together and in the broader context of international rural maternity care, however, consistency of findings indicates reliable evidence. The review team approached the research literature mindful of the importance of the role of context in the outcomes of the intervention (GPESS), in contrast to the more traditional cause-and-effect perspective. The focus on context was embedded in recommendations that were made based on the literature.

The commissioners convened two province-wide meetings of policy makers, practitioners, and administrators working in and with GPESS at which findings of this study were considered. The second meeting focused almost exclusively on the findings from the review. These meetings served as an “expert panel” for the review team and allowed a high degree of confidence that all relevant literature was included in the review.

## Results

**Fig. 1:**
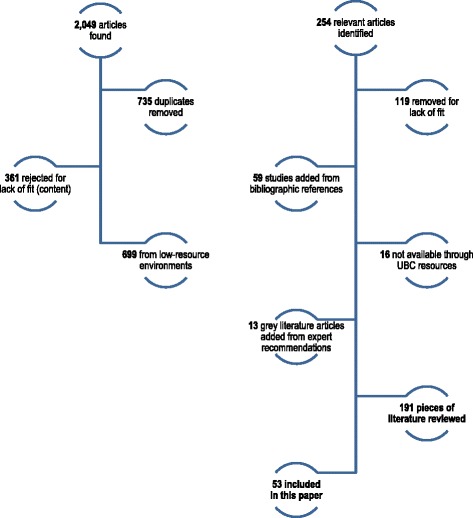
Exclusion Tree. The process of literature search exclusion

Two hundred fifty-four articles were originally identified as relevant from database searching. Upon consultation with the commissioners, one hundred nineteen articles were removed from consideration for lack of fit. These included articles highlighting clinical evidence on the relative safety of particular morbidities for parturient women (e.g. eclampsia, diabetes, HIV) (*n* = 27) and articles regarding defensive medicine and litigation concerns (*n* = 35). In total, 191 articles were subjected to in-depth review. Fifty-three pertained directly to safety and outcomes (see Fig. 1). A reference table is attached (see Additional file 1). A supplementary bibliography is also provided of those articles included in the full realist review but not specifically relevant to safety (see Additional file 2).

General practitioners with enhanced procedural skills have historically provided cesarean section support for rural maternity units in many jurisdictions internationally, including the United States [27, 40–48], Australia [49–52], Scotland [19, 53, 54], New Zealand [55], and across Canada [17, 22, 56–59]. Internationally, concern over volume thresholds and competency has ultimately led to a series of studies on the safety and service quality of GPs with Enhanced Surgical Skills. This research evidence can be categorized into five sub-categories: best practice standards, community expectations of safety, outcome comparisons by service provider, relationship of volume to outcomes, and consequences of small service closures. Each theme will be explored below.

### Best practice standards

Studies on best practices focus on either comparisons to published standards of practice or comparisons to specialist outcomes. One of the earliest contributions to this field is the retrospective chart audit at two rural hospitals in the US states of Washington and Oregon from, 1978–1992 by Deutchman et al. [60]. The authors found that GPs performed 79 % of cesarean section procedures at those hospitals. Reviewing the data from these deliveries, the authors concluded that GPs met or exceeded all standards of surgical outcomes in the published medical literature. An Australian study [61] reported on data on 5950 deliveries performed by GPs in rural New South Wales, Australia, between 1990 and 1991, and concluded that “[t]here is no evidence that obstetric care in NSW rural hospitals with accredited obstetric units is below standards acceptable to the community” (p242) when compared against all 88,275 deliveries in New South Wales in the same period.

International descriptive studies found similar results from GPESS-supported units. Kirke [62] looked at 195 births at a remote hospital with GPESS care 600 km east of Perth, Australia. Though complex and high-risk pregnancies were referred early, many women still in care went on to develop antenatal risk factors including hypertension, obesity, and pre-eclampsia, and the catchment population reported a high level of gestational diabetes. Intrapartum and post-partum complications such as maternal sepsis, antepartum hemorrhage, shoulder dystocia, failure to progress, and fetal distress occurred at rates similar to regional averages. No perinatal or maternal mortality was experienced in the study period, and health outcomes reported were as safe for mothers and babies as the specialist-led units. Cameron and Cameron [63] used obstetrical audit data from 1991–2000 at the GPESS-led rural Atherton hospital near Cairns, Australia, to show that perinatal mortality (stillbirth plus neonate death within 28 days) was substantially lower than the state average (5.3 per 1000 vs 11.8 for Queensland State or 11.8 for the Far North Queensland county). This unit was run by GPs, some of whom held an obstetrics diploma, with specialist support 96 kms away and access to outreach and evacuation services for only part of the study period. The community received four to six visits per year from specialist obstetrician-gynaecologists provided by the Far Northern Region Obstetrics and Gynaecology Service (FROGS).

In another Australian study, Scherman, Smith, and Davidson [64] studied the outcomes of a midwife-led unit with GP surgical support and OB specialist consultation in its first year (*n* = 164 births). The unit had low antenatal (10 %) and intrapartum (4 %) transfer, and 92 % spontaneous vertex delivery (i.e. 8 % intervention, including c-section, instrumental delivery, and breech birth). No Apgar scores below 7 were recorded at 5 min, and 89 % of neonates required no resuscitation. The rate of perinatal injury was half the state average at just 27 %. Though midwives led the unit, the authors contend that the low transfer rate was possible because of GP surgical support in the event of emergency.

### Comparison between levels of providers

A sub-set of the research reviewed compared GPESS-led services to specialist-led models. Aubrey-Bassler et al. [65] studied outcomes in four Canadian provinces (BC, Alberta, Saskatchewan, and Ontario), considering 1448 c-sections by 15 rural GPs and 4344 by specialists. Data was collected from Discharge Abstracts between 1991 and 2000, and showed that rates of iatrogenic morbidity were higher among GPs (OR 1.6; CI 1.1–2.3; 2.5 % vs. 1.6 % for specialists). However, this was accounted for by the difference in rate of puerperal infection (1.6 % vs. 0.8 % for specialists). Surgical error was the same between groups. GP proceduralists did, however, have higher rates of referral to acute care and their patients had longer post-surgical hospital stays (by 5.5 h on average).

These findings were echoed by Homan, Olson, and Johnson [16] in a smaller study between two comparable hospitals in New England. Using 125 consecutive c-sections from each hospital – one with GP-led maternal surgical care and the other with specialist-led surgery – this study found no difference in intraoperative or infectious complications, and no difference in neonate outcomes. Demographics of delivering mothers, prenatal risk factors, and indications for c-section were found to be similar between the two samples. The GP-led unit experienced fewer post-operative complications in contrast to the findings of Aubry-Bassler et al. [65], but the obstetrician-led unit did have a shorter post-operative stay.

Lynch et al. [66] compared two hospitals in British Columbia, one with c-section capability (Bella Coola) and one without (Haida Gwaii). In both communities, transfer or referral required considerable travel time and could be delayed by inclement weather. Between the two hospitals, there were no differences in adverse outcomes and no maternal deaths were reported in the study period (1986 to 2000) for either unit. The primary difference was in referral rates. Almost 20 % more local women were able to deliver in a c-section capable maternity unit than in the unit without surgical support due to the higher risk tolerance local operative service allows.

In the studies noted above, GPESS cases were pre-selected to include only low-risk courses of care with known complications referred to specialist obstetricians prior to delivery, diminishing the strength of findings. Using population level data addresses this methodological shortcoming, as demonstrated in the studies below.

The largest study of this kind in British Columbia examined 87,294 singleton births between 2000 and 2007. Grzybowski, Stoll, and Kornelsen [20] compared births from catchment areas with GPESS surgical support (*n* = 9,174) to the outcomes from obstetrician serviced catchments (*n* = 54,714). Using two-step logistic regression analysis to predict rates of adverse perinatal outcomes, the authors showed that health outcomes were comparable between GPESS-led surgical units, mixed-model units with both GPESS and specialists, and obstetrician surgical units. The authors found that 80 % of women delivered locally with GPESS support, while only 25 % could do so in communities without any surgical capability.

Iglesias et al. [22] used population data is their study of births in Alberta in 1999–2000, which examined patient outflow (the rate of patients leaving the community for care) and maternal-newborn outcomes based on level of local maternity services. The study illustrates that areas with limited maternity services are likely to have an increased rate of induction, and that in communities without local c-section capability there is large outflow. Communities that offered intrapartum care without local c-section capability delivered 22.1 % of the maternity population and this number increased to 70.1 % in communities with local c-section capabilities (level 1C).

Tucker et al. [19] found very similar rates in Europe’s most centralized health care system in Scotland. Comparing 1400 deliveries from eight of the twelve rural maternity catchments of Scotland, the authors demonstrated that roughly the same percentage of women remained “low-risk” throughout their pregnancy, and similarly, the rate of spontaneous vaginal delivery was stable when measured by catchment area rather than birth unit. Though low-risk cases were managed well by low-resource units, greater outflow from catchments with 1A equivalent services threatened sustainability. As with the Iglesias et al. [22] study above, midwife-only units (no surgical capability) were only able to perform 31 % of local deliveries, while midwife-led units with GP surgical support managed 70 % of local cases, and OB-led units performed 86 % of the births from their local catchments. Thus, the low intervention rates found in midwife-only and midwife-led units in other studies are shown to be reliant on referral and surgical support, as to be expected in a tiered service model with a risk management mandate.

Similar referral numbers appear in all population level data found for this review. Kornelsen, Grzybowski, and Iglesias [21] found that with GPESS support in a community, between 78 % and 85 % of births take place locally in BC and Alberta. Without c-section capability, that rate falls to between 24 % and 35 %. Humber and Dickinson [18] reported the most optimistic numbers, finding rates of 85 % and 40 % respectively.

### Service size and outcomes: is there a relationship?

Considerable attention is paid in the literature linking the size of maternity units with procedural outcomes, with some of the research evidence showing that the outcomes of small units are comparable to larger services. However, three studies indicate an outcomes *disadvantage* for small units, specifically among neonates.

A controversial study from Moster, Lie, and Markestad [67] found that Norwegian maternity units with 2000–3000 births per year had better outcomes than smaller units. This study looked at 700,000 low risk singleton births between 1972–1995 and found that units with <100 annual deliveries were almost twice as likely (OR 1.8; 1.1–3.1) to experience a late neonatal death (within 28 days of birth) than a unit with 2000-3000 births per year. However, the methodology of this study has limitations and several other studies undermine the power of many of the central claims by Moster, Lie, and Markestad [67].

Norum et al. [68] studied births from the scattered, northern, remote population of Norway and concluded that a very decentralized model of care that gave rise to smaller maternity units was necessary for a country where inclement weather and seasonal darkness makes transfer and even referral challenging. The pressing question is not whether the births that happened in higher level units were safer, but whether intrapartum care to women living in rural and remote areas would be safer and achieve better outcomes under centralized conditions. That is, when taking into account real-world, geographic constraints, what is the health cost of no local care? By excluding all out-of-hospital deliveries in their analysis, namely those that occurred during transfer, and by not considering the attendant challenges and health impacts of greater (or total) referral to centralized maternity units, Moster, Lie, and Markestad [67] avoid a critical geographic reality.

On the other hand, Viisainen et al. [69] examined accidental, out-of-hospitals births in Finland between 1962–1973, and compared them to data from 1992/93 (this data was not tracked in Finland between 1973 and 1992). Between 1962 and 1973, the rate of accidental, out-of-hospital birth fell from 1.3 per 1000 to 0.4 per 1000 whereas in 1992/93 it had reached 1.0 per 1000 live births. Viisainen et al. [69] argued there was a connection between the closure of small units and the rise in accidental, out-of-hospital births, events known to have exceptionally poor outcomes relative to delivery in hospitals. In fact, the crude risk factor for perinatal death was six times higher among babies born accidentally out of hospital, and over three times higher when birth weight is controlled [69, 70].

Despite increased concern over accidental, out-of-hospital births in Finland, the rate continued to increase during the 2000s according to Hemminki, Heino, and Gissler [70]. Their study of all births in Finland from 1991–2008 found that among children born weighing >2500 g (the same low-risk cut-off used by Mosler, Lie, and Markestad, [67] above), mortality was similar across all hospital types, sizes, and locations. However, the number of maternity units in Finland decreased 31 % over that span while births declined just 9 %, and accidental, out-of- hospital births increased. Of note, the rate normalized across regions during the study period, indicating that not just rural and remote women suffered this care deficit, but that urban-adjacent women also began to experience unplanned, out-of-hospital births in increasing numbers. This fits with data reported by Grzybowski, Stoll, and Kornelsen [6] from BC, Canada, that women between one and two hours from services were more than six times (OR = 6.41; CI 3.69–11.28) more likely to have an unplanned, out-of-hospital birth. Hemminki, Heino, and Gissler [70] provide a strong case for the need for smaller, local-to-mothers birthing units, concluding, “[t]he analysis suggests that in a regionalized system with a functioning referral system, there is no need to close down small hospitals for reasons related to health or healthcare procedures” (p1191).

Their conclusion echoes that of another Finnish study by Viisainen, Gissler, Hartikainen, and Hemminki [71]. Population birth data from 1987/88 was analyzed by service level of delivery hospital and catchment, selected for low-risk deliveries (*n* = 123,065). Their study showed good outcomes for all levels of service when low-weight and premature neonates and those requiring surveillance were cared for in hospitals providing the highest level of care (level 3). In a population catchment analysis [71], women determined to be low-risk had similar outcomes regardless of the hospital type at which they delivered; “[T]his study… indicates that ‘safety’ cannot be used as a basis for centralizing birth care in large level 3 facilities” (p404).

In a study done by Heller et al. [72], however, authors found a gradient of worsening outcomes from the largest and best resourced to the smallest birth units in Hesse, Germany. Looking at 582,655 births between 1990–1999, they reported that in units with <500 births per year, early neonatal death (within 7 days of birth) is three times more likely than in units with >1500 births annually. However, the authors note that without information on staffing, skill, training, levels of collaborative practice, and other indicators of quality of care within the delivery units, the influence of size of hospital in rates of higher mortality is unknown. Interestingly, this study uses the most inclusive definition of “low-risk,” calling all babies born of normal weight (2500 g–4200 g) without death by congenital abnormality a low-risk pregnancy and birth. Analysis that controlled for time of birth and gestational age and included late neonatal death (within 28 days) yielded similar results. In these analyses, however, maternal confounders were not controlled for.

Merlo et al. [73] also found a small unit outcome disadvantage, this time in Sweden, and attempted to define the percentage of proportional change in risk of neonatal mortality by birthing unit size. Using a multilevel logistic regression in which the outcomes of all births between 1990–1995 (*n* = 691,742) were nested in hospital level outcomes (*n* = 66), a confounder to hospital size was discovered. Just 4 % of Sweden’s institutionalized births take place in units with <500 annual births and without a pediatrics department, and this group showed the largest risk for neonatal mortality. The authors note, however, that the absolute survival rate in these relatively higher-risk birthing environments was 99.9 %, and the absolute survival difference compared to large regional hospitals was 0.06 % (or 0.6 deaths per 1000 births).

In response to these earlier studies, Tracy et al. [74] examined over 750,000 births over three years in Australia to compare outcomes by birthing unit annual volume. The study was limited to low-risk women. Among women without pre-existing or antenatal onset of hypertension or diabetes, and whose babies were born at >2500 g, rates of mortality were comparable in units with fewer than 100 deliveries and those with 2000 or more. Units of all sizes were found to have very similar outcomes, while smaller units tended to have less intervention, including lower rates of c-section [74]. Importantly, Tracy et al’s [74] categories for unit size and chosen sample size are in direct reference to Moster et al.’s [67] study, noted above.

Taken together, the differences in outcomes found by Heller et al. [72], Merlo et al [73], and Moster et al. [67] must be interpreted through a lens of *clinical* as well as statistical significance with attention paid also to potential iatrogenic costs due to lack of local access and travel. Further, the larger context of acceptable outcomes is important. Norum et al. [68] report a neonatal mortality rate of 2.2 per 1000 for all births in Northern Norway, and a national rate of 2.3 per 1000. For context, as of 2011, Germany also achieved a neonatal mortality rate of just over 2 deaths per 1000 births, roughly half of Canada’s rate of 4.7 [75]. Exceptional outcomes have already been achieved in small units from an international perspective, and the attendant health costs of greater centralization remain unknown in these three European studies.

Finally, there is a potential confound in the data of both Heller et al. [72] and Moster et al. [67]: the relative health of the adult population. Rural Canadians suffer a known health disadvantage compared to urban populations [76]. A study from Sweden by Finnstrom et al. [77] found lower rates of neonatal death, respiratory disturbance, cerebral palsy, and 5-min Apgar scores of <4 in smaller delivery units when controlling for maternal age, parity, gestational age, smoking during pregnancy, maternal body mass index, and parent cohabitation. Their massive study of 1.5 million singleton births between 1985 and 1999 found that in units with <500 annual births, the odds of neonatal death was just 0.84 (CI 0.63–1.11) compared to the reference category of units with 1000–2499 annual births [77], due in part to appropriate referral. Those units with 500–999 births did slightly better with an odds ratio of 0.82 (CI 0.73–0.92) of neonatal death. The authors found, as did Merlo et al. [73] above, that the existence of a pediatrics department played a significant role in lowering the neonatal mortality rate in smaller units, but the absolute numbers were too small to be statistically significant. They conclude that regionalized referral is functioning and that care is of a relatively homogeneous quality across unit size. These findings were validated in Sweden by Serenius et al. [78] when they examined the cause and context of all 9785 stillbirths and neonatal deaths in Sweden between 1983–1995. Again, data was controlled for maternal age, parity, and smoking during pregnancy, and again, the smallest units were found to be less likely to experience a death (OR = 0.65; CI 0.61–0.70). Efficient referral ensured that high-risk pregnancies were centralized to high-resource settings, while lower risk pregnancies showed strong outcomes when controlled for basic indicators of maternal health.

### Volume in relation to outcomes

The challenge of providing local access to cesarean section in rural settings rests in the low volume of procedures likely to be required among a low-risk population (assuming prior referral of parturient women with risk factors). The attendant concerns are regarding the maintenance of provider competency. However, volume-to-outcome associations are under-studied in Canada, and associations specific to maternal surgery are under-studied worldwide. In a review of volume-to-outcome association studies in the United States and Canada, Urbach et al. [79] found that Canada’s public health system considerably reduced the effect of volume on outcomes. Of 278 separate analyses reported in 142 articles reviewed by Urbach et al. [79], 206 (74 %) found a statistically significant association. Canadian studies were much less likely to find any association (OR = 0.24; CI 0.08–0.74). Though obstetrical specific data was collapsed into an “Other” category in Urbach et al.’s [79] analysis, even surgeries known to have a volume-to-outcome association (such as complex heart procedures) were shown to have a lesser effect intensity in Canada compared to the United States. The authors concluded that a single-payer, globally financed care system with regionalized organization reduces volume concerns, as complex procedures are already referred to high-level care facilities without inter-facility competition. However, only 14 of the 142 studies found by Urbach et al. [79] reported on Canadian data and just four of the studies included data on obstetrical procedures.

Using all births attended by family physicians at BC Women’s Hospital and Health Centre from 1997–1998 (*n* = 4,444 births), Klein et al. [80] analyzed outcomes according the personal volume of attending family physicians (*n* = 152 physicians). Thresholds of <12, 12–24, and >25 were used to explore whether attending more births led to better birth outcomes, but no differences were found in the volume cohorts in maternal complications, 5-min Apgar scores <7, or adverse admissions to intensive or special care. Low-volume GPs were more likely to consult with an obstetrician and more likely to transfer care to a specialist, but outcomes were not affected by attending a lesser volume of births.

### Distance matters

Examining 49,402 births to women from rural catchments between 2000–2004, Grzybowski, Stoll, and Kornelsen [6] found that neonatal mortality was three times more likely for births in which the women had to travel four or more hours to services (OR = 3.17; CI 1.45–6.95). As well, induction was found to be 1.3 times more likely in women who had to travel to services, mostly for logistical reasons [81].

Even in the relatively more dense Netherlands, longer travel times are associated with worsened outcomes [82]. Travel of more than 20 min to care resulted in higher total mortality (OR = 1.17; CI 1.002–1.36), higher neonatal mortality within 24 h (OR = 1.51; CI 1.13–2.02), and greater rates of adverse outcomes (OR = 1.27; CI 1.17–1.38) in Ravelli et al’s [82] study of 751,926 births in Holland between 2000–2006. Few women in the Netherlands travel more than 30 min (as measured by driving time without delays) to birthing services, which contrasts with the geographic realities of BC. However, their finding of an odds ratio of additional risk of 1.01 (CI 1.00–1.01) per minute of travel time corroborates the findings of Grzybowski, Stoll, and Kornelsen [6] above. Though no one in the Netherlands would have to travel four hours (240 min) to service, by extrapolating Ravelli et al’s [82] per-minute findings, the increased risk of neonatal mortality for such a long travel time would be OR = 3.40 – just slightly higher than the 3.17 number found here in BC. Such a finding from a very different health context is evocative when considering the centralization of services as a method of improving outcomes.

## Discussion

Research literature has shown that local access to cesarean section increases the proportion of women safely able to deliver in their local community to at least 70 % from 30 % in services not offering local cesarean section capacity. Finding and supporting the health human resource compliment in communities with enough volume to sustain such services, however, has been difficult. The very nature of rural services is defined by low volume, making specialist practice in the smaller communities unfeasible. The solution in the international jurisdictions covered in this review has been a reliance on GPs with Enhanced Surgical Skills. Due to the number of sites supporting GP procedural practice and the number of evaluative studies that have resulted, research evidence on the safety and efficacy of this practice is strong. Perhaps equally as importantly, there is no existing clinical, case study or qualitative evidence to suggest that cesarean section is less safe when provided by a GPESS than when provided by a specialist obstetrician.

Supporting and sustaining local maternity services is crucially important to achieving good perinatal health outcomes. Although the proportion of outflow from the community is reduced with local operative delivery, research evidence also tells us that the lack of any local maternity service is worse than services without cesarean section. This is due in part to the unintended morbidities incurred when women present to an unprepared service fully dilated, or physiological and psycho-social morbidities, caused by travelling to access care. Additionally, health service realities – including the lack of continuity of care when women leave their communities – must be accounted for in a comprehensive review of safety of the evidence on small local surgical services.

Enmeshed in concerns over the safety of the practice of GPESS, there has also been the ongoing debate on *practice thresholds*; that is, the number of procedures performed, both by individual clinicians or in facilities, in relation to outcomes. The literature in this review suggests that volume-to-outcomes associations are extremely variable across procedure and context, but as a whole greater birth volume does not improve birth outcomes. This does not speak to greater *procedural* volume, however, specifically in regards to cesarean sections. Although we do know that greater volume increases confidence (particularly greater volume in residency) [36], careful consideration of the relationship between GPESS volume of cesarean sections and outcomes is a crucial gap in our evidence and in need of further investigation.

Although a context-mechanism-outcomes (CMO) theory was not postulated at the onset of this review due to the pragmatic intent of the commissioners, it is clear that an *a posteriori* understanding of CMO can be understood from the reviewed literature and applied to the creation of evidence-based models of care.

The *context* for these models must include a statement of support from a governance level recognizing the importance of meeting the perinatal surgical needs of rural women as close to home as possible, respecting complexity of procedure, risk status of patient, and health conditions in the community. Additionally, surgical care should be viewed as a regional, rather than institutional, phenomenon. Consequently, the scope of practice and resources needed to implement surgical programs should be organized regionally. General Practitioners with Enhanced Surgical Skills (GPESS) can be safely allowed to practice to the fullest extent of their ability within the context of a regionalized and inter-professional system of referral, consultation, and emergency transfer support. Small ORs should become outreach extensions of core referral hospital surgical programs, and the organization of services should respect the sustainability of the regional referral services and the smaller services.

The *mechanism* needed to enact the vision of continuous perinatal surgical services (24/7 c-section backup) includes services provided through a well-integrated and balanced surgical team, which includes outreach surgeons and local generalist surgical providers. Surgical competency could be enhanced by regular rotation of team members through a larger referral centre. Training programs for rural nurses need to be strengthened, recognizing the broad skillset and multifaceted nature of rural nursing. Small service surgical team skills and competencies should be built and maintained through an integrated educational program with local referral hospitals. This can be accomplished both through outreach and by rotating small service surgical team members through the referral community’s surgical program. Additionally, inter-professional outreach surgical educational and mentorship activities extended from the regional referral hospital to the small surgical sites on a regular basis. This model requires timely and regular feedback within a quality improvement framework.

Anticipated *outcomes* of the context and mechanism described include a robust and sustainable networked model of rural surgical services equipped to meet basic perinatal surgical needs of rural populations.

## Limitations

Following the indications of realist reviews to privilege context in the structure of the review and the interpretation of findings, this review is directly applicable to the history, context and political challenges in British Columbia, Canada. Although similar conditions may be found in other jurisdictions, the exact constellation of conditions will not be the same, thus limiting transferability to other settings.

This review was commissioned in response to a real-time planning challenge that arose out of a provincial priority-setting initiative informed by external time-lines. Because of this, a rapid review approach was used. This demanded attention to the balance of *comprehensiveness* and *timeliness*. Although methodological rigor was applied throughout the process, the exhaustiveness of the search could be potentially limited due to time constraints. This was addressed through the involvement of the expert panel, made up of key clinically, politically, and administratively engaged stakeholders in the province. This scrutiny of the review allowed room for the inclusion of grey literature references not captured through the search strategy.

## Conclusion

Clinical, case study, and qualitative evidence demonstrates that perinatal surgical care is equally safe when provided by GPESS and specialist physicians. This finding allows health planners to confidently build perinatal surgical services around the contribution of GPs with Enhanced Surgical Skills and focus on educational, regulatory, and continuing professional development mechanisms to ensure their sustainability. Volume-to-outcomes associations are variable and inconclusive with regards to safety, suggesting both the need for more evidence and also the viability of low-volume services particularly suited to generalists who can take on other roles in the community. These findings, and the attendant health services planning directions, are reassuring as they suggest the viability of local models of care where feasible. This policy direction addresses the social and health risks to women from communities without local access to maternity care, leading to improved health outcomes.

## Additional files

Additional file 1: Article Reference Chart. Details of each of the 53 articles from the full realist review considered for its contribution to safety and outcomes and included in this manuscript. (PDF 305 kb) Additional file 2: Supplementary Bibliography. All the articles included in the full realist review are detailed here. (PDF 192 kb)
