# Supplementary material for: Centralized or decentralized perinatal surgical care for rural women: a realist review of the evidence on safety
Source: BMC Health Serv Res. 2016 Aug 13;16:381. doi: 10.1186/s12913-016-1629-6 (PMC4983412; doi:10.1186/s12913-016-1629-6)
Supplement: Additional file 1: — Article Reference Chart. Details of each of the 53 articles from the full realist review considered for its contribution to safety and outcomes and included in this manuscript. (PDF 305 kb) [file 12913_2016_1629_MOESM1_ESM.pdf]

Bibliographic reference	Jurisdiction	Research question	Context	Study Design	Main Findings
Aubrey-Bassler, K., Newbery, S., Kelly, L., Weaver, B., & Wilson, S. **Maternal outcomes of cesarean sections: do generalists' patients have different outcomes than specialists' patients?** Canadian family physician. 2007. **53(12)**: 2132-8.	Small, isolated hospitals in Ontario, BC, Alberta, Saskatchewan	• How do maternal outcomes of Cesarean section compare when performed by a GP versus a specialist?	• Rural and semirural GP surgeons in Canada being compared to specialists in high resource environments<br>• Authors note that rurality could be its own impacting variable: rural patient population likely to be lower SES and have higher parity rate; potential for limited care in rural areas; staff possibly less aware of operating room best practices	Retrospective matched cohort	• Significantly higher rates of all major morbidity and major surgical morbidity were found among GPs. However when, major postpartum infection was excluded there was no difference.<br>• GPS safety not only within expected guidelines, but comparable to obstetrics specialists in low-risk populations
Baker DK: **Rural surgery in Canada**. *World Journal of Surgery* 2006, **30(9)**: 1632–3.	Rural Ontario	• What are the challenges faced by rural surgeons in Ontario?	• Rural surgeons not often prepared for challenges of position due to lack of specific focus on training rural providers<br>• Patient safety is compromised by long travel in emergencies and inclement weather<br>• Author argues that Canadian rural surgery is dying off slowly because of lack of support/recognition for its particular challenges	Editorial written by 1 of the 17 rural surgeons in Ontario	• Author recommends a commitment to rural surgery as a specialty in Canadian medical schools, better locum support from provincial medical associations, and more support for continuing education<br>• Also suggests a model of care in which touring surgical teams come into communities to do elective cases, leaving GP Surgeons to do emergency operations
Breon TA, Scott-Conner CEH, Tracy RD: **Spectrum of general surgery in rural Iowa.** *Current surgery* 2003, 60(1): 94–9.	Rural Iowa	• What types of surgery are performed by rural surgeons?<br>• How do the practices of urban and rural surgeons differ in Iowa?	• Surgical Need:<br>• In 1996, 31 general surgeons recruiting partners, 25 of them rural<br>• 1996: 18 rural hospital administrators actively recruiting surgeons<br>• Most positions had been open over a year, most still open in 2000<br>• Of 1,000 surgical residents graduating annually, only 25% go in to general surgery, and only 10% of those work in rural areas (25 of 1,000 annual US graduates)<br>• Existing shortage compounded by expected increase in general surgical services	Cross-section survey and qualitative interviews	• Existing need in surgical work force large and growing<br>• General surgeons declining in favour of subspecialties, with the risk that maldistribution results alongside shortage<br>• Incentives for rural and general surgery have to be developed/introduced or reinforced

			• 25% of people (55 Million) in US live in rural areas, just 12.3% of physicians work in rural areas<br>• 1988: urban population had physician to population ratio of 448:1, rural 1038:1<br>• In counties with fewer than 25,000, ratio was 1639:1<br>• In Iowa, ratio exceeds 3000:1 in ¼ of rural counties<br>• Nationally, just 10% of general surgeons work in rural areas. Estimates for projected need in 2000 was that 19% would need to work in rural areas for adequate care<br>• Rapid closure of rural hospitals		
Callaghan, J. **A twenty-five year survey of a solo practice in rural surgical care**. Journal of the American College of Surgeons. 1994, **178(5)**: 459-65.	Small rural hospital in Decorah, Iowa	• What are the surgical care and surgical outcomes of a rural hospital in Iowa?	• Rural residents of this study prefer to be treated in local hospital than neighboring medical centre<br>• The strong social connection between patients and health care professionals has suggested higher patient satisfaction and better patient care.	Hospital-based retrospective cohort study	• The range of surgical treatment expected from the surgeon in a rural setting exceeds that of the surgeon in a metropolitan or large medical center. Action should be taken to ensure adequate training for those who wish to practice in the rural setting.
Cameron B: **Outcomes in rural obstetrics, Atherton Hospital 1981-1990**. *The Australian Journal of Rural Health* 1998, **6(1)**: 46–51.	The Atherton Hospital, Far North of Queensland (rural hospital)	• How to maternal and newborn outcomes from Atherton Hospital compare to outcomes from Queensland and the Far North Statistical Division?	• Although the paper did not address directly, it is implied that decentralized model is in favor in geographically isolated area where there is a lack of access to specialist service as the perinatal mortality rate at Atherton Hospital compared favorably with Queensland and the surrounding Far North Statistical Division figures.	Retrospective cohort study	• This study found that maternal and newborn outcomes in this small rural hospital compared favourably to regional and provincial outcomes. This suggests that maternity care in a rural hospital can be safe where antenatal and obstetric care is provided a GP.
Cameron B, Cameron S: **Outcomes in rural obstetrics, Atherton Hospital 1991-2000**. *Aust J Rural Health* 2001 **9(1)**:39–42.	The Atherton Hospital, Far North of Queensland (rural hospital)	• How to maternal and newborn outcomes from Atherton Hospital compare to outcomes from	• There is a decline in private rural obstetricians who serve as vital support backup for their public hospital colleagues. This suggests that it is important that salaried Medical Officers have adequate	Hospital-based retrospective cohort study	• Perinatal mortality at Atherton Hospital: 5.3/1000 vs. Far North Queensland region 11.8/1000 vs. Queensland: 11.3/1000<br>• No maternal deaths<br>• The C-section rate at Atherton Hospital has

		Queensland and the Far North Statistical Division?	levels of obstetric training.		raised to 17.4% during 1991-2000 from 13.0% during 1981-1990<br>• Good perinatal and obstetric outcomes occur at small rural hospitals where obstetric care is provided by the non-specialist rural doctor<br>• The perinatal mortality has remained relatively stable over the two decades
Campbell NA, Kitchen G, Campbell IA: **Operative experience of general surgeons in a rural hospital**. *ANZ Journal of Surgery* 2011, **81(9)**: 601–3.	Wimmera Base Hospital in northwestern Victoria, Australia (rural hospital)	• With what frequency, and under what conditions did the two surgeons at Wimmera Base Hospital encounter various surgical procedures over a five year period?	• Lack of surgeons in rural areas of Australia and difficult to attract surgeons to rural areas<br>• The authors note that there has been a succession of physicians with varying degrees of expertise at Wimmera Base Hospital during the study period<br>• The residents need to travel 200km to the nearest larger hospital	Retrospective review of surgical logbooks	• Surgical trainees intent on practicing in rural areas should seek to gain experiences appropriate to meeting the needs of the community where they intend to practice. A broad skill base is required, and should include the ability to manage surgical emergencies across all specialties. A supportive team of medical and paramedical staff is required for achieving the best outcome for patients.
Deutchman M, Connor P, Gobbo R, FitzSimmons R: **Outcomes of cesarean sections performed by family physicians and the training they received: a 15-year retrospective study.** J Am Board Fam Pract 1995, **8(2)**:81–90.	Two rural hospitals: one in Oregon and one in Washington	• How do rates of obstetric intervention and outcomes compare for two rural hospitals over a period of 10-15 years?	• Due to the distance to the nearest tertiary care centre, referring patients to obtain obstetric care is often impractical	Retrospective cohort study	• About 79% of all c-sections at the 2 study hospitals were performed by family physicians as the primary surgeons. Only 2.2% were performed by an obstetrician/gynecologist<br>• C-sections carried out my family physicians is not a common practice in the United States. The authors mentioned that only 4.5% of active members of the American Academy of Family Physicians have cesarean privileges at their hospitals.<br>• The medical chart and logbook review showed that c-sections completed by family physicians at rural regional hospitals were relatively safe with few reported complications<br>• This suggested that residency-trained, board-certified physicians can help to support obstetric needs at rural hospitals
Dooley J, Kelly L, St Pierre-Hansen N, Antone I, Guilfoyle J, O'Driscoll T: **Rural and remote**	Sioux Lookout Meno Ya Win Health	• What outcomes are associated with the small rural health centre,	• 2007 report created by the Society of Obstetricians and Gynecologists of Canada identified a need for to increased opportunities and reduce	Program evaluation	• Comparable caesarean deliveries are made in the program as compared to the provincial averages.<br>• Success rate of vaginal birth after

**obstetric care close to home: program description, evaluation and discussion of Sioux Lookout Meno Ya Win Health Centre obstetrics.** *Can J Rural Med* 2009, **14(2)**:75–9.	Centre Program. Northern Ontario	Sioux Lookout Meno Ya Win Health Centre Program?	barriers for Aboriginal women to deliver close to home in a familiar environment.<br>• Closures of obstetrics programs and decreasing physician availability in rural areas are deemed by Ontario Women's Health Council as a maternity crisis.<br>• Recently, the BC government has allocated funds to re-establish family physician involvement in obstetrics.		caesarean was 80% versus the provincial success rate of 53%.<br>• Gestational diabetes rates resulted in 25.5% rate of large-for-gestational-age babies versus a provincial rate of 11%.<br>• No intrapartum foetal or maternal death and only 1 readmission (2006-2008)<br>• 6 new-borns weighing less than 2500 g were delivered.<br>• Programs such as this need a volume of 300-350 deliveries per year. If not, they need funding for physicians and nurses to visit regional centers to maintain competence
Finnström O, Berg G, Norman A, Olausson PO: **Size of delivery unit and neonatal outcome in Sweden. A catchment area analysis.** *Acta Obstet Gynecol Scand* 2006 **85(1)**:63–7.	Sweden, National	• What is the relationship between delivery unit volume and neonatal outcomes?	• Comparing 1.5 million singleton births between 1985-1999, authors found improved neonatal outcomes over the study period. Neonatal mortality declined from 3.3 per 1,000 deliveries to 2.1 in the 15 years under study.<br>• Of 67 maternity units in 1985, 7 small units closed during study period, as did 2 of the largest units. 4 further units were merged with neighboring units, leaving 54 units in 1999.<br>• The proportion of mothers in catchments served by the smallest delivery units (<500 annual births) who actually delivered there was ~80%	Retrospective Cohort Study	• Units with <500 births per year had fewer deaths, better Apgar scores, and fewer respiratory issues on average than units with 1000-2500 births when maternal health controlled<br>• However, neonates from catchments served by small maternity units had on average slightly higher neonatal mortality (not statistically significant) even with maternal health controlled<br>• Better neonatal outcomes in small delivery units shows effective regionalization, while worsened outcomes for rural catchments is not explained.<br>• Small units without a pediatrics department had much worse outcomes than those with such a department, suggesting factors beyond volume have to be considered<br>• Authors conclude that quality of care is homogeneous across size of delivery unit and that regionalization is functioning appropriately
Grzybowski S, Stoll K, Kornelsen J: **Distance matters: a population based study examining access to maternity**	British Columbia, Canada	• How does distance required to travel to access the nearest maternity services with	• Current policy change toward centralization has externalized financial and health costs of worsened maternal outcomes, while downloading direct costs to health	Retrospective cohort study	• Perinatal mortality increases by factor of 3.17 for families traveling >4hrs<br>• Increased rates of NICU 2 admission and more NICU bed days for mothers 1-2 hours from service

**services for rural women.** *BMC Health Serv Res* 2011, **11**:147.		Cesarean section capability impact maternal and newborn outcomes?	care users. The effects are both clinical (as discussed below) and social, as rapid centralization has reduced access to high quality care for many women.		• Much higher rate of birth en route for women living 1-2 hours from services<br>• Higher rates of induction for logistical reasons in women without local care facilities<br>• Higher rates of maternal intervention under care by specialist
Grzybowski S, Stoll K, Kornelsen J: **The outcomes of perinatal surgical services in rural British Columbia: a population-based study.** *Can J Rural Med* 2013,**18(4)**:123–9.	Provincial study- British Columbia, Canada	• What is the relative safety of GPESS attended births to those at other service levels?	• Rural maternity services are being discontinued in British Columbia and across rural Canada, often<br>• because of the loss of local surgical services. General Practitioners with enhanced surgical skills can provide operative backup for perinatal surgical care in rural catchment areas that are too small to support specialist surgeons because they can provide generalist primary care as the core part of their practices.	Retrospectiv e population-based cohort study	• The population outcomes for small surgical services staffed by GPESSs were as good as the population outcomes for referral services staffed by obstetricians.
Heller G, Richardson DK, Schnell R, Misselwitz B, Künzel W, Schmidt S: **Are we regionalized enough? Early-neonatal deaths in low-risk births by the size of delivery units in Hesse, Germany 1990-1999.** *Int J Epidemiol* 2002 **31(5)**:1061–8.	Hesse, Germany	• Is the size of delivery units associated with early-neonatal death among low-risk births?	• There is little evidence for the benefits of regionalization of maternity care for low-risk births	Population-based retrospectiv e cohort study	• Birthweight-specific mortality rates were highest in the smallest delivery units and lowest in the largest delivery units<br>• The mortality rates however were low for all size categories among low-risk births (6 per 1000 for the smallest centres and 1.9 per 1000 for the largest centres)
Hemminki E, Heino a, Gissler M: **Should births be centralised in higher level hospitals? Experiences from regionalised health care in Finland.** *BJOG* 2011 **118(10)**:1186–95.	Finland	• What are the trends in centralization and unplanned out-of-hospital births?<br>• Is place of birth associated with perinatal mortality?<br>• What health and	• Maternal care in Finland is universal, and pre and post natal care are decentralized and run by local communities<br>• Births are part of specialist care	Hospital-based retrospectiv e cohort study	• For normal birth weight babies, there was no difference in mortality rates by hospital size<br>• The perinatal mortality rate for unplanned out-of-hospital births was seven times higher than that for hospital births<br>• These findings do not support the closing of small rural hospitals in a regionalized system with a functioning referral system

		birth outcomes are associated with hospitals of different levels of service?			
Homan FF, Olson AL, Johnson DJ: **A comparison of cesarean delivery outcomes for rural family physicians and obstetricians.** *J Am Board Fam Med* 2013, **26(4)**:366–72.	2 rural community hospitals in New England, USA - one where cesarean sections are performed by a family physician (FMH), the other where cesarean sections are done by an obstetrician (OBH)	• How do outcomes of caesarean sections performed by family physicians compare to those performed by obstetricians in two comparable rural hospitals?	• The number of family physicians who perform cesarean deliveries in the United States has declined, as has the availability of obstetricians in many underserved areas.<br>• Well-trained family physicians report difficulties in securing hospital privileges to do cesarean deliveries.	Retrospective chart review	• Patients did not have increased risk when cesarean delivery was performed by a family doctor rather than an obstetrician.<br>• Rates of intraoperative complications and infectious complications were similar for both hospitals. Neonatal outcomes were similar, and there were no differences in pre-term deliveries.<br>• There were fewer postoperative complications at the FMH than the OBH.
Hueston WJ, Murry M: **A three-tier model for the delivery of rural obstetrical care using a nurse midwife and family physician copractice.** *J Rural Health* 1992, **8(4)**:283–90.	Kentucky, USA	• Describe the formation and operation of a hospital-sponsored nurse midwife and family physician co-practice.	• Unavailability of local prenatal care creates hardship and stress for women who are most likely to develop complications of pregnancy.<br>• Over the past decade, family physicians have started to stop obstetrical practice due to concern over malpractice, adequacy of obstetric training and lifestyle issues.<br>• The primary reason cited by midwives for not locating to rural areas was the lack of physicians to serve as their sponsors; thus a cooperative venture of nurse midwives and family physicians would not work.	Hospital-based retrospective chart review	• Evidence shows that the maternity center patient's received care comparable to other patients at the St. Claire Medical Center.<br>• There was a small increase in the percentage of newborns who were cared for in the special care nursery; however, this increase was not statistically significant and may be secondary to the decline of patients who needed to be transferred to tertiary care centers.<br>• The three-tier model of care outlined utilizes obstetricians for the tasks that are most consistent with their training, i.e. surgical intervention and consultation in the care of the high risk pregnancy. Thus, this system is more cost effective than employing obstetricians to care for low-risk

					women.
Humber N, Dickinson P: **Rural patients' experiences accessing surgery in British Columbia.** *Canadian Journal of Surgery* 2010. p. 373–8.	Lillooet Health Area, a rural community in BC operating with one single GP-surgeon and GP-anesthesiologist	• How does the presence or absence of rural surgical services influence patients psychosocially?	• Over the decade of 2000-2010, the delivery of surgical services became increasingly centralized. Small-volume surgical sites (5,000-20,000 residents) that were traditionally supported by family doctors with enhanced surgical skills decreased by nearly 50% in British Columbia.<br>• This was driven by the aim to optimize cost- and resource-effectiveness, volumes, and outcomes, but does not consider the qualitative emotional, psychosocial, and cultural impacts of these closures on patients and communities.<br>• Lillooet Health Area is one of the more socioeconomically disadvantaged local health areas in BC. Figures for the number of individuals receiving income assistance, as well as alcohol consumption, are double the provincial average.	Case study	• Rural patients are different than their urban counterparts; a rural population has different challenges in accessing health care and its own disparities and determinants of health. Multiple shared experiences create a unique doctor-patient relationship in rural areas. This relationship influences the way rural residents make health care decisions.<br>• To all rural residents interviewed in this study, receiving care in their own communities from familiar health care providers took precedence over perceived quality of surgical care.
Humber N, Frecker T: **Delivery models of rural surgical services in British Columbia (1996-2005): are general practitioner-surgeons still part of the picture?** *Canadian Journal of Surgery* 2008a, **51(3)**: 173-178.	British Columbia; rural care areas with no general surgeon or specialist support, where General Practitioner Surgeons are the primary surgical care providers	• How have models of surgical service delivery in rural areas changed over the past decade?<br>• What influence does the loss of surgical services have on a rural community?	• Communities of less than 15,000 people cannot recruit and maintain specialist surgeons, relying instead on GP-Surgical care for both elective and emergency surgery<br>• There is no specific training program for rural surgical specialists/rural general or GP surgeons	Retrospective study	• "Communities that wish to consistently provide full-service maternity care to 85%–90% of women but are more than 100 km from a referral centre need to maintain a local surgical program." (p. 175)<br>• Even with closures (25% loss of GP only surgery sites in BC between 1996 and 2004), GP Surgeons continue to play important role in rural acute care, emergency care, and obstetrics/maternal surgical care<br>• A training program for GP surgeons is needed to replace aging workforce before rural surgical services are lost
Iglesias A, Iglesias S, Arnold D. **Birth in Bella**	Bella Bella, British	• What were the factors surrounding	• Due to the specialization of family practitioners in primary care,	Retrospective cohort	• Perinatal and maternal mortality rates, as well as rates of intervention were

**Bella: emergence and demise of a rural family medicine birthing service.** Canadian family physician ca; 2010. p. e233–40.	Columbia	the closure of the once successful rural maternity care program?	secondary services, including anesthesia and surgery, became part of the scope of the Royal College of Physicians and Surgeons of Canada<br>• Access to training in procedural skills became increasingly difficult for rural physicians<br>• The maternity service in Bella Bella had been supported by generalist physicians for nearly a century. But closed in 2001 due to difficulties in recruiting generalist physicians and physicians who would provide intrapartum services	study	comparable to Canadian data<br>• When the skillset of family doctors changed, rural communities suffered because of the inability to recruit doctors with the necessary skills to sustain rural maternity services
Iglesias, S., Bott, N., Ellehoj, E., Yee, J., Jennissen, B., Bunnah, T., & Schopflocher, D. **Outcomes of maternity care services in Alberta, 1999 and 2000: a population-based analysis.** Journal of obstetrics and gynaecology Canada, 2005, 27(9): 855.	Alberta, Canada	• How do maternal and perinatal birth outcomes compare for communities with limited or no local intrapartum care to those with regional and tertiary care?	• Only 22.1% of women from communities with limited maternity care programs (without CS capability; 1A) delivered in their home community<br>• This contrasts to a study by Black and Fyfe (1984) that found women in Northern Ontario delivered at home at a rate of 57-80% when without CS capability. Authors argue that this is a clear sign of centralization in Canadian maternity services over the 20 year period between studies.<br>• Authors note that while regionalization has created equitable results across various levels of service provision, increased centralization threatens to undermine that. Communities without local services are possible only because of nearby communities with 1A and 1C services.	Population-based retrospective study	• Authors contend that services in any given community have to be considered within the eco-system of care as a whole. Though good health outcomes have been found in communities without Cesarean section, and even those without any local service, increased centralization could have cascading effects on outcomes and travel remains problematic.
Johnson D, Jin Y: **Low-volume obstetrics. Characteristics of family physicians'**	Alberta, Canada	• How do rates of obstetric intervention and	• The Society of Obstetricians and Gynecologists of Canada has taken the position that physicians are not	Retrospective cohort study	• The authors speculate that LVFPs have been replaced by HVFPs in smaller hospitals and that high-volume family

**practices in Alberta.** *Canadian family physician* 2002, **48:** 1208–15.		birth outcomes compare for low-volume family practice (LVFP) and high-volume family practice (HVFP)?	required to participate in a certain number of deliveries per year to maintain competence; however they recommend that those who attend fewer than 25 births/year should be restricted to low-risk practice and should update their skills.<br>• The SOGC also recommends that the standard of care for low risk maternity patients should be equivalent to that in tertiary care centres as in small rural hospitals.<br>• The number of rural and remote hospitals that offer obstetric services has decreased over the last decade and centralization has occurred and many remote and rural communities are left with no local access to maternity care services.		practice does not result in higher-risk obstetrical practice.<br>• High-risk women are usually cared for by obstetricians in urban centers and thus even when HVFPs offer local obstetric services, they are not always used.<br>• The closing of small hospitals would have detrimental effects on rural and aboriginal women.<br>• The authors suggest that 25 deliveries/year is arbitrary and there is no evidence that suggests that quality of care and volume are directly linked.
Kirke AB: **How safe is GP obstetrics? An assessment of antenatal risk factors and perinatal outcomes in one rural practice.** *Rural and remote health* 2010 **10(3)**:1545.	Kalgoorlie, Western Australia	• What outcomes are associated with a small rural obstetric practice run by nonspecialist GP obstetricians?	• Nearly one quarter of pregnant women in Western Australia are from rural or remote regions, and about 80% of women living in these areas deliver locally. The medical workforce attending to these patients is predominantly made up of general practitioner obstetricians.<br>• The challenge is for GP obstetricians to quickly refer high-risk patients to an appropriate facility, and to develop and maintain the skills necessary to adequately deal with whatever else occurs.	Hospital-based retrospective chart review	• The isolated and scattered nature of rural health provision often means that there are fewer resources per person in rural and remote populations than those in urban areas. However, rural people still need access to adequate and appropriate health care. This study supports that argument that obstetric care can be safely provided by appropriately trained general practitioners in areas where specialist services are less accessible.

Klein MC, Spence A, Kaczorowski J, Kelly A, Grzybowski S: **Does delivery volume of family physicians predict maternal and newborn outcome?** *CMAJ* 2002 **166(10)**:1257–63.	BC Women's Hospital and Health Centre, Vancouver, BC	• Do the practice-volume relations that have been shown in other fields of medical practice also exist in maternity care practice by family doctors?	• Better outcomes have been associated with higher patient volumes in some instances, but not others. Most of the studies on volume have focused on surgical or oncology specialties.	Cross sectional Analysis-Physicians grouped into 3 categories based on # of births the attended each year (low, medium, high volume)	• Family Physicians delivery volumes were not associated with adverse outcomes for mothers or newborns.<br>• Low-volume family physicians referred patients and transferred deliveries to obstetricians more frequently than high- or medium-volume family physicians.
Kornelsen J, Grzybowski S, Iglesias S. **Is rural maternity care sustainable without general practitioner surgeons?** Canadian journal of rural medicine. 2006. p. 218–20.	Canada	• Is rural maternity care sustainable without general practitioner surgeons?	• Many rural maternity services in North America have closed and the consequences are not well understood<br>• Maternity services without cesarean section capability are vulnerable to closure<br>• Studies have reported psychosocial costs to women who do not have local access to maternity services<br>• Canada has not actively pursued an approach to train, certify and provide quality assurance for GP surgeons (as has occurred for GP anesthesia and advanced maternity skills)	Expert opinion piece	• Evidence suggests that GP surgeons are critical to the sustainability of rural maternity and surgical services<br>• There are significant challenges for GP surgeons to access local training and mentorship<br>• There is a current lack of acknowledgement of the importance of GP surgeons in rural areas
Kornelsen J, Moola S, Grzybowski S: **Does distance matter? Increased induction rates for rural women who have to travel for intrapartum care.** *J Obstet Gynaecol Can* 2009, **31(1)**:21–7.	Provincial study- British Columbia, Canada	• How do intervention rates and outcomes differ between women who live adjacent to maternity service with specialist (surgical) services	• In Canada, between 3% and 23.5 % of parturient women undergo induction of labour. Although Canadian data for<br>• calculating the rate of inductions that are not performed for medical or obstetrical indications are limited, international research indicates that there is significant variation in	Cross-sectional study	• Rural parturient women who have to travel for care are 1.3 times more likely to undergo induction of labour than women who do not have to travel. Further research is required to determine why this is the case.

		and women who must travel for this care?	induction rates between hospitals in th same jurisdiction, indicating that multiple, contextual variables affect the decision to induce labour.		
Kozhimannil KB, Law MR, Virnig BA: **Cesarean Delivery Rates Vary Tenfold Among US Hospitals; Reducing Variation May Address Quality and Cost Issues.** Health Aff 2013, 32(3):527–35	United States- National Study	• How can we reduce the large variation of Cesarean section rates among US hospitals to address important health and cost implications?	• In the US, caesarean section increased from 20.7 % of all deliveries in 1996 to 32.8 % in 2011. Caesarean delivery is much more costly than vaginal delivery, and is also associated with worse outcomes and complications for women.	Cross-sectional study	• It was found that rates of caesarean section in US hospitals vary 7.1% to 69.9%. This large variation indicates that clinical risk factors probably do not provide a full explanation for these differences. It is likely that practice patterns are a likely driver of variations in delivery mode and ought to be the focus of policy interventions to slow or reverse the rise in caesarean delivery rates overall and to decrease variation across hospitals.
Larimore WL, Davis A: **Relation of infant mortality to the availability of maternity care in rural Florida**. *J Am Board Fam Pract* 1991, **8(5)**:392–9.	Rural counties in Florida	• What is the impact of the availability of maternal care services on infant mortality rates in rural counties in Florida?	• Forty-seven counties in Florida were lacking in maternity care services at the time of the study.	Cross-sectional study; hypothetical model	• Access to maternity care in rural Florida is a problem that could be hampering Florida's ability to reduce its infant mortality rate.<br>• Family physicians appear to be the most geographically distributed health care providers in Florida; therefore, strategies should be developed to recruit Florida's rural family physicians into maternity care.
Leeman L, Leeman R: **A Native American community with a 7% cesarean delivery rate: does case mix, ethnicity, or labor management explain the low rate?** Ann Fam Med 2003, 1(1):36–43.	Predominantly Native American region of northwestern New Mexico, USA.	• Can studying communities with low rates of cesarean delivery help identify practices that can lower the cesarean rate?	• Cesarean delivery rates can vary widely among different population groups; known risk factors include age, parity, weight, socioeconomic status, ethnicity, and other obstetrical risk factors. Studying communities with low rates of cesarean delivery may help to identify practices that lower the cesarean delivery rate. This study focused on births at the Zuni-Ramah Hospital to identify factors associated with the low c-section rate of 7.3% in this predominantly Native American population.	Population-based cohort study	• The low rate of caesarean delivery in the Zuni-Ramah community can be attributed to a number of factors including, but not limited to: the predominant involvement of family physicians and nurse-midwives attending births, lower birth weights, and cultural attitudes towards childbirth.

Leeman L, Leeman R: **Do all hospitals need cesarean delivery capability? An outcomes study of maternity care in a rural hospital without on-site cesarean capability.** *J Fam Pract* 2002, **51(2)**:129–34.	Zuni Pueblo and Ramah Navajo communities of North-western New Mexico (predominantly Native American region)	• What birth outcomes are associated with maternity care services provided at a small rural hospital without Cesarean section capability?	• Guidelines for Perinatal Care developed by the American College of Obstetricians and Gynecologists and the American Academy of Pediatrics include the statement "all hospitals that offer labor and delivery should be able to perform emergency cesarean deliveries".	Population-based retrospective cohort study	• Perinatal outcomes and care in rural communities produce better outcomes than systems that require women to travel to distant urban areas for maternity care.<br>• The study purports that the use of oxytocin in rural hospital units without operative facilities should be considered under well-defined clinical guidelines or research protocol. Moreover, it calls for guidelines to be developed to permit rural hospitals without cesarean capability to provide maternity care as part of integrated perinatal systems with well-develops transport protocols and supportive referral institutions.
Lisonkova S, Sheps SB, Janssen PA, Lee SK, Dahlgren L, Macnab YC: **Birth outcomes among older mothers in rural versus urban areas: a residence-based approach**. *J Rural Health 2011*, **27(2)**:211–9.	British Columbia	• Are maternal and newborn birth outcomes among older mothers associated with rural residence?<br>• How do parity and distance to nearest hospital influence the association between rural residence and birth outcomes?	• In the developed world, delayed childbirth has become increasingly common<br>• It is unknown if outcomes differ for older women based on geographic barriers to accessing advanced obstetric care	Population-based retrospective cohort study	• Among older women, the cesarean section rate was lower and there was an increased risk of perinatal mortality in rural compared to urban areas
Lynch N, Thommasen H, Anderson N, Grzybowski S: **Does having cesarean section capability make a difference to a small rural maternity service?** *Can Fam Physician* 2005, **51**:1238–9.	Bella Coola General Hospital (with cesarean section capability) in Bella Coola Valley, BC; Queen	• How does access to Cesarean section in an isolated rural community influence rates of adverse maternal and newborn birth outcomes?	• The practice of obstetrics in rural Canadian communities is changing, towards offering less and less obstetric care in the form of anesthesia, epidural, and cesarean section.<br>• Bella Coola Valley Hospital had cesarean section capability throughout most of 1986-2000. Queen Charlotte Islands General Hospital offered obstetric services,	Retrospective cohort study	• Comparing local births only, there was no difference between both hospitals in rates of episiotomy for vaginal delivery or adverse outcomes.<br>• No maternal deaths were reported in either population.<br>• There was a significantly higher rate of premature delivery at the Queen Charlotte Islands General Hospital (without cesarean capability) than the Bella Coola General Hospital. This was not explained by

	Charlotte Islands General Hospital (without cesarean section capability) in Queen Charlotte City, BC.		but not cesarean section deliveries. Both hospitals are otherwise similar in population size, Northern and Isolation Allowance program designation, type of hospital, and availability of local obstetric services.<br>• The referral hospitals closest to Bella Coola Valley are in Williams Lake (more than 450 km by road) or in Vancouver (2-hour flight). The referral hospital closest to Queen Charlotte City is in Prince Rupert (6-hour ferry or 2-hour float plane trip); the nearest centre with obstetricians and pediatricians is in Vancouver (4-hour flight). For both centres, inclement weather can complicate transport.		differences in ethnicity. This increased prematurity is likely linked to increased outflow.<br>• Over the study period, almost 20% more women were able to remain in their home communities to deliver when local operative delivery was available
Lynge DC, Larson EH, Thompson MJ, Rosenblatt R a, Hart LG: **A longitudinal analysis of the general surgery workforce in the United States, 1981-2005**. *Arch Surg* 2008, **143(4)**:345–50.	United States-National Study	• How can we explain the decline in the number of general surgeons in the USA over the past two decades?	• The overall supply of general surgeons per 100,000 population has declined in the past two decades, and small and isolated rural areas of the United States continue to have relatively fewer general surgeons per 100,000 population than urban areas.	Retrospective longitudinal analysis	• The overall number of general surgeons per 100,000 population has declined by 25.91% during the past 25 years.<br>• The decline has been most marked in urban areas, but more remote rural areas continue to have significantly fewer general surgeons per 100,000 population. These findings have implications for training, recruiting, and retaining general surgeons.
Lynge DC, Larson EH: **Workforce issues in rural surgery**. *Surg Clin North Am* 2009 **89(6)**:1285–91, vii.	United States-National Study	• How are rural surgeons distributed among rural and urban areas in the United States?	• Although the overall proportion of gernal surgeons working in rural areas is approximately proportional to the population of rural America, closer examination reveals significant maldistribution of general surgeons across regions and different types of rural areas.	Expert Opinion	• Large rural areas are well supplied with general surgeons compared to urban or isolated rural areas. Small or isolated areas may have only two to three general surgeons per 100,000 population, and many small town and rural hospitals have no surgeons at all. There is a need for a number of recruitment strategies and initiatives to make rural surgery more attractive to the modern surgical graduate.
Merlo J, Gerdtham U, Eckerlund I, Håkansson S, Pakkanen M, Lindqvist	Sweden	• How do rates of neonatal mortality differ by hospital	• For high-risk births, studies have reported increased survival rates when maternity services are	Retrospective population-	• From a medical point of view the findings suggest that the regionalization of birth for low-risk pregnancies is justified (ie. The

P, et al: **Hospital Level of Care and Neonatal Mortality in Low- the Question in Sweden by Multilevel Reassessing Analysis**. *Med Care* 2014, **43(11)**:1092–100		level of care in low and high risk births?	regionalized to concentrate resources in larger hospitals<br>• Some recent studies have suggested that regionalization is also beneficial for low-risk deliveries	based cohort study	regional centres had decreased mortality rates)<br>• From a public health perspective, the closure of small maternity unit may prevent a noticeable number of deaths, however this change would not make a substantial difference at the individual level due to the low neonatal mortality rate in Sweden
Moster D, Lie RT, Markestad T: **Relation between size of delivery unit and neonatal death in low risk deliveries: population based study.** *Arch Dis Child Fetal Neonatal Ed* 1999, **80(3)**:221–5.	Norway	• Is the size of a hospital delivery unit associated with neonatal mortality for low risk pregnancies?	• There is no consensus on the optimal conditions for low risk pregnancies<br>• As there are no antenatal screening procedures that can ensure an uncomplicated delivery, it can be argued that a well-equipped hospital is the safest place for all deliveries<br>• Alternatively, low-risk deliveries may be at increased risk for medical attention when they may benefit from limited intervention	Retrospective population-based cohort study	• The risk of neonatal death increased as the number of deliveries at an institution decreased to less than 2000/year<br>• The risk of neonatal death was double in hospitals with less than 100 deliveries/year compared to hospitals with more than 3000<br>• However, the neonatal mortality rate for low risk pregnancies is extremely low irrespective of birth place
Nesbitt, TS, Connell, FA, Hart, FA, and Rosenblatt, RA: **Access to Obstetric Care in Rural Areas: Effect on Birth Outcomes** *American Journal of Public Health* 1990, **80(7)**:814–18.	Rural area of Washington State	• How do birth outcomes compare for women residing in low-outflow communities (where >2/3 of deliveries occurred in the local hospital) and women residing in high-outflow communities (where <1/3 of deliveries occurred in the local hospital?)	• A decline in the number of physicians offering routine obstetrical care in US<br>• In rural areas, the number of physicians offering routine obstetrical care drop by 23% to 43% since 1980<br>• The proportion of rural physicians who offered obstetrical care is higher than national average (43% vs. 29%)<br>• This decline was largely due to the cost of liability insurance and the feat of law suits<br>• Women from areas with limited obstetrical service had to travel long distance to obtain basic prenatal care and delivery	Hospital-based retrospective cohort study	• Women living in rural Washington state communities with little or obstetrical care availability locally tend to deliver in hospitals outside the community. These women are more likely to have complicated labor and premature deliveries, and their infants are more likely to have longer and more expensive hospitals than the children of their rural counterparts who deliver in local facilities communities with greater access to care.<br>• The authors suggested that the long travel distance to hospitals with obstetrical services may be a barrier associated with poorer prenatal compliance, particularly for low income women or women without adequate transportation.<br>• Birth associated complications in high-outflow communities may be due to delays in presentation to the hospital after the onset of labor and increased physiological

					and psychological stress associated with traveling long distance to unfamiliar settings.
Norum J, Heyd A, Hjelseth B, Svee T, Murer FA, Erlandsen R, et al: **Quality of obstetric care in the sparsely populated sub-arctic area of Norway 2009-2011.** *BMC Pregnancy Childbirth* 2013, **13**:175	Norway	• How do birth outcomes in northern Norway (decentralized care) compare to all of Norway?	• Northern Norway has a decentralized model of maternity care with midwives providing care for lowest risk women<br>• Intermediate risk women deliver in local hospitals and women with high risk deliver in regional hospitals<br>• There are no obstetricians in the midwife administered maternity units, however there is a general practitioner on duty in the community<br>• The Ministry of Health and Care Services recently launched a plan to improve maternity services which would involve centralizing care	Population-based retrospective cohort study	• Women in Northern Norway received a similar quality of care as Norwegians in general<br>• There were significantly more low birth weight newborns in Northern Norway
Pollett W, Harris K: **The future of rural surgical care in Canada: a time for action.** *Can J Surg* 2002, **45(2)**: 88-89.	Canada (rural and remote)	• What is the future of rural surgical care in Canada?	• Impending crisis in surgical care in Canada, most imminently in rural and remote areas where general surgeons are aging/retiring and there is no system to replace them with other broadly trained surgeons who are willing to work in rural conditions	Report	• Recommendations:<br>• Immediately increase the # of training positions for general surgeons<br>• Recruit for rural placements early in the training process such that a physician's training can be tailored to community-specific needs<br>• Recognition of rural/community surgery as a distinct specialty with academic infrastructure from Royal College of Phy & Surg of Canada, University depts and specialist societies to train this group<br>• Role for GPs and other health care providers to take on a limited amount of surgical procedures if these providers have appropriate certification from the Royal College or its equivalent<br>• Develop regional centres/networks that provide appropriate infrastructure and critical mass of cases to maintain competence<br>• Locum support to address lifestyle issues
Ravelli a CJ, Jager KJ,	The	• What is the effect	• Access to maternity care is	Population-	• A travel time of greater than 20 minutes

de Groot MH, Erwich JJHM, Rijninks van Driel GC, Tromp M, *et al*: **Travel time from home to hospital and adverse perinatal outcomes in women at term in the Netherlands**. *BJOG* 2011, **118(4)**:*457–65*	Netherlands	of travel time during labour from home to hospital on perinatal death and adverse outcomes?	decreased in rural areas and travel times to hospital may be longer<br>• Women in the Netherlands often stay at home until the signs of labour are obvious to the midwife, contrary to other countries where women are admitted to the hospital upon the first signs of labour	based cohort study	from home to hospital is associated with statistically significant increased risk of mortality and adverse outcomes<br>• This finding should be considered alongside centralization of maternity services
Robinson M, Slaney GM, Jones GI, Robinson JB: **GP Proceduralists: "the hidden heart" of rural and regional health in Australia**. *Rural Remote Health* 2010, **10(3)**:1402.	Bogong region, Australia	• What is the extent, type, and frequency of procedural medicine in the area?<br>• What is the estimated supply and demand for the GP proceduralist workforce in the future?	• Small rural towns throughout Australia are dependent on GP's and GP proceduralists (GP's who do one or more of obstetrics, anesthetics, and surgery) for procedural and after-hour medical services.<br>• In rural Australia, there is a trend towards retirement and an increasingly poor GP: population ratio.<br>• Even as medical studies intake increases, the increase unlikely to meet the doctor demand due to changing demographics and community expectations as well as part-time work preferences for doctors and a preference towards a decreased work hours/week.<br>• Centralization of services has resulted in many small hospital closings; between 1983 and 2005 in Victoria, 72% of small rural obstetrics units were disbanded.	Qualitative case study	• If GPs skills decline because of lowering volume and decreasing complexity, their confidence will decrease potentially lading to adverse outcomes or necessity to travel long distances for patients.<br>• Declining services in rural settings shifts the costs to larger hospitals and reduces the availability in rural regions.<br>• Health sector has changed both nationally and internationally; however, the need for basic care in rural and remote communities has not.<br>• Even if one GP proceduralists leaves a rural, there is a detrimental effect to the rest of the system.<br>• The future of procedural practice in the Bogong region depends on numerous factors including: demand, sufficient interest of new doctors, and funding.
Roos N, Black C, Wade J, and Decker K: **How Many General Surgeons Do You Need in Rural Areas? Three Approaches to Physician Resource Planning in Southern Manitoba**. *Canadian*	Southern Manitoba, Canada	• In comparing three models of surgeon allocation, how many general surgeons are needed in rural Manitoba?	• The three models of physician resource planning are:<br>• The ratio model which uses a population to surgeon ratio,<br>• The repatriation model which is based on the number of patients currently leaving their community to access surgery, and the number of	Retrospective review of medical charts	• Authors note that rural south has an average number of elderly, a typical rate of pre-mature (before 74) death, have low socio-economic risk, and an above average state of health. Thus, they may be exposed to a higher rate of surgery than is necessary. The obvious implication for health care planning is that the region doesn't need to add general surgeons.

Medical Association Journal 1996, **155(4)**: 395-400.			surgeons that could be supported by the region, and<br>• The population-needs-based model which looks at the age and health structure of the community and the current rate of surgery.		• Authors further argue that needs-based and repatriation approaches are the most meaningful as they are based in the actual number of surgeries being performed.<br>• Authors argue that overlap in who does procedures makes ideal ratios misleading, as many general surgery procedures are already being done by non-general surgeons.<br>• Authors argue the need for resource planning that examines population characteristics known to be related to the need for care
Scherman S, Smith J, Davidson M: **The first year of a midwifery-led model of care in Far North Queensland**. *Med J Aust* 2008, **188(2)**:85–8.	Mareeba (64km southwest of Cairns), Far North Queensland, rural Australia	• What birth outcomes were associated with the first year of rural midwifery-led model of care?	• Community had maternity unit in community hospital, with 196 deliveries per year on average from 2000-2004<br>• 2005 unit closed due to inability to recruit sufficiently skilled personnel<br>• Six weeks later, unit re-opened led by midwives<br>• At that point, Cairns hospital became referral maternity surgical ward	Hospital-based prospective cohort study	• Raises potential of hierarchical care model where primary care is delivered by midwives without surgical training<br>• Of 158 women to deliver at midwife-led unit, 146 (92%) had spontaneous vaginal delivery<br>• Rate of emergency lower segment c-section was 1.2% including intrapartum transfers (n=2)<br>• A further 4.4% (7) elective LSCS (total c-section of 5.6%)<br>• 2004 Queensland State average of all LSCS is 30.7%<br>• Rate of perinatal injury almost half of state average: Total injury rate = 27%. State Avg = 55.3%<br>• 1.3% episiotomy (state avg=12.4%); 0% 3rd/4th degree (1.1% avg); 8.7% 2nd degree (21.3%); 17.3% 1st degree (20.5%).<br>• Analgesia use also roughly half of state average: 66.7% took none, compared to 36.4% state average<br>• Average apgar scores of delivered babies at midwife led unit were 8 at 1 minute, 9 at 5 minutes. None had a score of less than 7 at 5 minutes. 89% required no resuscitative measures

					• Concern that some women are presenting at midwifery unity despite high risk categorization and being advised to deliver at the referral hospital in Cairns
Serenius F, Winbo I, Dahiquist G, Källén B: **Cause-specific stillbirth and neonatal death in Sweden: a catchment area-based analysis.** *Acta Paediatr* 2001, **90(9)**:1054–61	Sweden	• How do rates of stillbirth and neonatal mortality differ for geographical area of mother's residence grouped by degree of hospital specialization?	• The development of intensive neonatal care has improved the survival rates of very small newborns<br>• Studies have reported an effect of hospital size on the survival of preterm infants<br>• However, it can be argued that the reduced facilities in small hospitals can be compensated for by referral of high risk pregnancies<br>• A catchment based analysis using the mother's area of residence is needed to study this question	Population-based cohort study	• Total mortality was not increased when comparing areas with the lowest level of neonatal care to areas with the highest level of care<br>• There was a difference when the analysis was repeated for cause-specific deaths, specifically death due to obstetric complication (which increased with decreasing level of care of the catchment hospital)
Simmers D: **The few: New Zealand's diminishing number of rural GPs providing maternity services.** *New Zealand Medical Journal* 2006, **119(1241)**	New Zealand	• Can the New Zealand maternity service afford to lose the services of general practitioner obstetricians?	• There are 54 general practitioner obstetricians providing intrapartum services in New Zealand<br>• The role that the general practitioner obstetricians play in supporting rural maternity services must be recognized<br>• Evidence supports rural women to give birth in their own communities	Commentar y	• Planning is underway for a specialist vocational training program for rural hospital doctors<br>• This program should include a maternity skill set<br>• Training in technical skills that have been considered secondary care, as well as the ability to know when to refer care, are needed for rural primary maternity care providers
Tracy SK, Sullivan E, Dahlen H, Black D, Wang YA, Tracy MB: Does size matter? **A population-based study of birth in lower volume maternity hospitals for low risk women.** *BJOG* 2006, **113(1)**:86–96	Australia	• How do birth outcomes for low risk women differ by volume of hospital births per year?	• Small maternity hospitals are closing in Australia and internationally based on the belief that lower-volume hospitals may have a decreased quality of care<br>• For normal-weight babies of women who have an uneventful pregnancy, it is unknown where the safest place to birth is<br>• There is a balance between the need for safety and the preservation of primary level birth facilities	Population-based cohort study	• Lower hospital volume is not associated with adverse birth outcomes for low risk women
Tucker J, Hundley V,	Scotland	• What are the	• Scant evidence about the quality of	Cross-	• Rural professionals, including midwives in

Kiger A, Bryers H, Caldow J, Farmer J, et al: **Sustainable maternity services in remote and rural Scotland? A qualitative survey of staff views on required skills, competencies and training**. *Qual Saf Health Care* 2005 **14(1)**:34–40.		staff's views on required skills, competencies, and training?	care in remote and rural acute maternity services<br>• 30% of Scotland's population lives in rural areas	sectional study	dual and triple duty posts, must maintain a broad range of skills as generalists.<br>• Medical coverage appears increasingly unsustainable due to the current trend towards subspecialization which makes general practice more difficult<br>• Staff in rural hospitals reported sufficient competence and confidence to perform maternity services. The quality of local services is threatened due to the loss of medical cover rather than a lack of staff preparedness.<br>• Tele-health technologies were reported as being rarely used, but enthusiasm was shown for the possibility of using it to offset the reduced access to care in rural areas
Tucker, J., McVicar, A., Pitchforth, E., Farmer, J., & Bryers, H. **Maternity care models in a remote and rural network: assessing clinical appropriateness and outcome indicators**. *Qual Saf Health Care*, 2010, **19**(**2**), 83-89.	Remote and rural maternity units in North of Scotland	• How do service levels, clinical appropriateness and birth outcomes compare for 3 rural staffing models of care?	• Scotland was said to be among Europe's most centralized systems of maternity care (Wildeman et al., 2003)<br>• At the same time, concern exists that centralization of obstetric and neonatal services is limiting access of rural and remote women to intrapartum care<br>• Staffing in small hospitals is difficult to sustain<br>• Tiered services, including midwife led intrapartum care for low risk women has been recommended by the National Service Framework in England and Wales, the Framework for Scotland, and the Expert Group on Acute Maternity Services in Scotland	Population-based retrospective cohort	• The findings describe a health care model in which the needs of rural women are the same regardless of what care is available (to be expected) and in which services without surgical support are far less likely to provide local birthing options in a risk management policy environment<br>• The authors call for further research into the lower rate of c-section and higher rate of NNU >48hrs among births from low-service catchments.
Urbach DR, Croxford R, MacCallum NL, Stukel T: **How are volume-outcome associations**	The United States and Canada	• How are volume-outcome associations	• Many studies have found that surgical outcomes are better when done by hospitals or providers who	Systematic literature review	• Canadian analyses were substantially less likely to find a statistically significant volume-outcome association than US studies

**related to models of health care funding and delivery? A comparison of the United States and Canada**. *World J Surg* 2005, **29(10)**:1230–3		related to models of health care funding and delivery?	do them more frequently—which has led to the regionalization of certain procedures<br>• Some studies suggest that the volume-outcome associations are artifacts of underlying variation in hospital outcomes<br>• Little research has been conducted to examine how health care delivery and financing affect procedure volumes, outcomes and volume-outcome associations		
Viisainen K, Gissler M, Hemminki E: **Birth outcomes by level of obstetric care in Finland: a catchment area based analysis.** *J Epidemiol Community Health* 1994 **48(4)**:400–5	Finland	• What is the incidence and geographical distribution of accidental out-of-hospital births in Finland?<br>• How do perinatal outcomes from accidental births compare to hospital births?	• Many small rural maternity units have closed as a consequence of centralization of maternity services<br>• Centralization results in an increase in travel time and therefore an increased risk of birth in transit or accidental home birth	Retrospective cohort study	• The birthweight adjusted risk of perinatal death was significantly higher in accidental births than in hospital births (OR 3.11, CI 1.42-6.84)<br>• There was in increase in accidental births in the 1990s that correlates with the closing of small hospitals<br>• Centralization policies in sparsely populated areas should include measures to prevent accidental out-of-hospital births
Viisainen K, Gissler M, Hartikainen a L, Hemminki E: **Accidental out-of-hospital births in Finland: incidence and geographical distribution 1963-1995.** *Acta Obstet Gynecol Scand* 1999, **78(5)**:372–8	Finland	• Are hospitals with different levels of maternity care equally safe places to give birth in a regionalized system of care?	• The question of safety of small primary maternity hospitals has provoked the closing of many small maternity hospitals<br>• The benefits of tertiary care for low-birth weight babies has been shown, however for normal birth weight babies studies have indicated that outcomes at small primary centres are the same, if not better, than tertiary centres	Cross-sectional study	• In a regionalized system of care with proper referral systems in place, small local hospitals are as safe for childbirth as tertiary care hospitals
Wadland WC, Havron AF, Garr D, Schneeweiss R, Smith M: **National survey on hospital-based privileges in family practice**	United States	• What are the regional variations of hospital-based privileges among members of the AAFP?	• Maternal and neonatal health indicators are worsening in the US despite spending more money per capita on maternal and neonatal care than any other developed country	Cross-sectional study	• Obstetrical care by family physicians is one possible solution to the growing inadequacy of maternal and neonatal care<br>• Rural physicians were significantly more able to perform the three most restrictive procedures: vaginal delivery with forceps,

**obstetrics**. *Archives of family medicine* 1994, **3(9)**: 793–800.			• The US ranks 16th in the world in infant mortality, most likely due to poor accessibility to primary prenatal care<br>• Family physicians who continue to offer obstetrical care usually provide full prenatal and intrapartum care. Family physicians who stop caring for pregnant women tend to give up all obstetrical care and never resume<br>• Due to the large-scale withdrawal of family physicians from maternity care, it is important to know what hospital-based privileges FPs can obtain		c-section, and amniocentesis<br>• Most physicians (>95%) were satisfied with their level of privilege<br>• A considerable number of obstetrical privileges are granted to family physicians, but there is no uniformity in privilege due to regional variation.<br>• Teaching hospitals reportedly restrict obstetrical care by family physicians more than other hospitals<br>• In larger hospitals where specialists are available, privileges in obstetrics for family physicians are more limited
Welch R, Power R: **General practitioner obstetric practice in rural and remote Western Australia**. *Aust New Zeal J Obstet Gynaecol* 1995, **35(3)**:241–4.	Western Australia	• How can we describe the practice of obstetrics by General Practitioners in rural and remote areas of Western Australia?	• One-quarter of women confined in Western Australia hospitals during 1992 were confined in country hospitals. Rural and remote general practitioners are responsible for providing obstetric services to the majority of women living in non-metropolitan communities. It has been argued that obstetric practice in rural and remote areas is just as safe as in the city.	Cross-sectional study	• Of all GPs currently practicing obstetrics in rural and remote areas, 90% were male. GPs in rural and remote areas reported an average of 28 deliveries per year, and had lower rates of performing Caesarean section, using forceps and ventouse compared to Western Australia as a whole. Reasons for GPs decision to cease practice of obstetrics include feat of litigation and increasing medical insurance premiums.
Woollard LA, Hays RB: **Rural obstetrics in NSW**. *Aust N Z J Obstet Gynaecol* 1993, **33(3)**:240–2.	New South Wales, Australia	• How does the quality of intrapartum care in rural hospitals compare to non-rural standards?	• Rural GPs are responsible for providing obstetric services to the majority of rural women	Cross-sectional survey and retrospective cohort study	• Authors note that because of referral patterns of high-risk patients, rural delivery outcomes cannot be said to be better than metropolitan<br>• Still, the overall health and safety are considered "good" by the authors<br>• In light of so many current GPs being interested in training future GP obstetricians, authors suggest that more training should take place in rural units.
