# Supplementary material for: Centralized or decentralized perinatal surgical care for rural women: a realist review of the evidence on safety
Source: BMC Health Serv Res. 2016 Aug 13;16:381. doi: 10.1186/s12913-016-1629-6 (PMC4983412; doi:10.1186/s12913-016-1629-6)
Supplement: Additional file 2: — Supplementary Bibliography. All the articles included in the full realist review are detailed here. (PDF 192 kb) [file 12913_2016_1629_MOESM2_ESM.pdf]

1. Albers LL, Savitz DA: **Hospital setting for birth and use of medical procedures in low-risk women**. *Journal of nurse-midwifery* 1991, 36(6)327–33.
2. Alexander C: **Why doctors would stay in rural practice in the New England health area of New South Wales**. *Aust J Rural Health* 1998, **6(3)**:136–9.
3. Allen DI, Kamradt JM: **Relationship of infant mortality to the availability of obstetrical care in Indiana.** *Journal of Family Practice* 1991, **33(6)**:609.
4. Anderson G: **The effect of a rural track residency program with an emphasis on obstetrics on physician shortage areas.** *Journal of Rural Health* 2000, p. 230–1.
5. Angle P, Kurtz Landy C, Murthy Y, Cino P: **Key issues and barriers to obstetrical anesthesia care in Ontario community hospitals with fewer than 2,000 deliveries annually.** *Canadian Journal of Anesthesia* 2009, **56(9)**:667–77.
6. Australian College of Midwives: **Costing caesarean sections: a pilot study.** *Australian College of Midwives* 1994, **7(2)**: 6–10.
7. Backe B: *Maternity Care in Norway***.** Norwegian University of Science and Technology, National Advisory Committee for Obstetrics. http://www.helse-nord.no/getfile.php/RHF%20INTER/Prosjekter/Making%20it%20Work/Maternity%20care%20in%20Norway%20-%20Bjorn%20Backe.pdf
8. Baker JW, Buttini MJ: **The Flying Obstetric and Gynaecology Service in rural Queensland: Its first two years.** *The Medical Journal of Australia 1991*, **154(9)**: 578–82.
9. Bar-Zeev J. S, Barclay L, Farrington C, Kildea S: **From hospital to home: The quality and safety of a postnatal discharge system used for remote dwelling Aboriginal mothers and infants in the top end of Australia**. *Midwifery* 2012, **28(3)**:366–73.
10. Baumann A, Hunsberger M, Blythe J, Crea M: **Sustainability of the workforce: government policies and the rural fit.** *Health Policy* 2008, **85(3)**:372–9.
11. Belsky D, Ricketts T, Poley S, Gaul K, Fraher E, Sheldon G: *Surgical Deserts in the US : Places Without Surgeons.* American College of Surgeons Health Policy Research Institute; 2009. http://www.acshpri.org/documents/ACSHPRI_FS2.pdf
12. Black C, Burchill C: An assessment of the potential for repatriating care from urban to rural Manitoba. *Medical Care.* 1999, **37(6)**: 167–186.
13. Borgstrom DC, Heneghan SJ: **Bassett Healthcare rural surgery experience**. *Surgical Clinics of North America.* 2009. **89(6)**: 1321–ix.
14. Bourke L, Humphreys JS, Wakerman J, Taylor J: **From "problem-describing" to "problem-solving": challenging the "deficit" view of remote and rural health**. *Aust J Rural Health* 2010, **18(5)**:205–9.
15. Buck ST, Trauba V, Christensen RG: **Minnesota physician workforce analysis: rural supply and demand.** *Minnesota medicine* 2004, **87(9)**: 40–3.
16. Burkholder HC, Cofer JB: **Rural surgery training: a survey of program directors**. *J Am Coll Surg* 2007, **204(3)**:416–21.
17. Buser KB: **Laparoscopic surgery in the pregnant patient--one surgeon's experience in a small rural hospital.** *JSLS : Journal of the Society of Laparoendoscopic Surgeons* 2002, **6(2)**: 121–4.
18. Cameron B: **Outcomes in rural obstetrics, Atherton Hospital 1981-1990**. *The Australian Journal of Rural Health* 1998, **6(1)**: 46–51.
19. Campbell DG, Greacen JH, Giddings PH, Skinner LP: **Regionalisation of general practice training: are we meeting the needs of rural Australia**? *Med J Aust* 2011, **194(11)**: S71:S74
20. Canadian Family Physicians: **Joint position paper on training for rural family practitioners in advanced maternity skills and Cesarean section.** *Can Fam Physician* 1999, **(45)**: 2416–32.
21. Chan FY: **Fetal tele-ultrasound and tele-therapy**. *Journal of telemedicine and telecare* 2007, **13(4)**: 167–71.
22. Chang Pecci C, Leeman L, Wilkinson J: **Family medicine obstetrics fellowship graduates: training and post-fellowship experience**. *Family Medicine* 2008, **40(5)**: 326–32.
23. Chaytors RG, Szafran O, Crutcher RA: **Rural-urban and gender differences in procedures performed by family practice residency graduates.** *Family Medicine* 2001, **33(10)**:766–71.

24. Cheng YW, Snowden JM, Handler S, Tager IB, Hubbard A, Caughey AB: **Clinicians' practice environment is associated with a higher likelihood of recommending cesarean deliveries.** *J Matern Neonatal* 2013
25. Cogbill TH, Cofer JB, Jarman BT: **Contemporary Issues in Rural Surgery.** *Current Problems in Surgery* 2012, **49(5)**: 263–318.
26. Cross DA: **Guidelines for regional anesthesia in obstetrics: Potential impact on obstetric anesthesia services in rural Alabama.** *South Med J* 1993, 86(1):33–7.
27. Crump WJ, Fricker RS, Ziegler C, Wiegman DL, Rowland ML: **Rural track training based at a small regional campus: equivalency of training, residency choice, and practice location of graduates.** *Acad Med* 2013, **88(8)**:1122–8.
28. Cyna AM: **Anaesthesia in rural Queensland: clinical experience with the Flying Obstetric and Gynaecology Service**. *Anaesth Intensive Care* 1993, **21(6)**:831–6.
29. Davies PG: **Problems with training for general practice in South Australia**. *Med J Aust* 1991, 155(7):457–62.
30. Doty B, Heneghan SJ, Zuckerman R: **General surgery contributes to the financial health of rural hospitals and communities**. *Surg Clin North Am* 2009, 89(6):1383–7, x–xi.
31. Doty B, Zuckerman R: **Rural surgery: framing the issues**. *Surg Clin North* 2009, **89(6)**:1279–84, vii.
32. Doty B, Zuckerman R, Finlayson S, Jenkins P, Rieb N, Heneghan S: **General surgery at rural hospitals: a national survey of rural hospital administrators**. *Surgery* 2008, **143(5)**:599–606.
33. Drew J, Cashman SB, Savageau JA, Stenger J: **The visiting specialist model of rural health care delivery.** *J Rural Health* 2006, **22(4)**:294–9.
34. Eley DS, Synnott R, Baker PG, Chater AB: **A decade of Australian Rural Clinical School graduates--where are they and why?** *Rural Remote Health* 2012, **12**:1937.
35. Farmer J, Lauder W, Richards H, Sharkey S: **Dr John has gone: assessing health professionals' contribution to remote rural community sustainability in the UK.** *Soc Sci Med* 2003 57(4):673–86.
36. Gaff-Smith M: **Are rural adolescents necessarily at risk of obstetric and birth outcomes?** *Australian Journal of Rural Health* 2005, **13(2)**: 65-70.
37. Gates RL, Walker JT, Denning DA: **Workforce patterns of rural surgeons in West Virginia.** *Am Surg* 2003: **69(5)**:367–71.
38. Gillman LM, Vergis A: **General surgery graduates may be ill prepared to enter rural or community surgical practice**. *Am J Surg* 2013, **205(6)**:752–7.
39. Glasser M, Peters K, Macdowell M: **Rural Illinois hospital chief executive officers' perceptions of provider shortages and issues in rural recruitment and retention**. *J Rural Health* 2006, **22(1)**:59–62.
40. Glazebrook RM, Harrison SL: **Obstacles and solutions to maintenance of advanced procedural skills for rural and remote medical practitioners in Australia**. *The Journal of Rural Health* 2006,**6(4)**:502.
41. Government of Western Australia - Department of Health: *WA Health Clinical Services Framework 2010-2020*. 2010. http://www.health.wa.gov.au/hrit/docs/clinicalframework.pdf
42. Gruen RL, Knox S, Britt H: **Where there is no surgeon: the effect of specialist proximity on general practitioners' referral rates.** *Med J Aust* 2002, **177(2)**:111–5.
43. Grzybowski SC: **Problems of providing limited obstetrical services to small, isolated, rural populations**. *Can Fam Physician* 1998, **44**:223.
44. Grzybowski SC, Cadesky AS, Hogg WE: **Rural obstetrics: a 5-year prospective study of the outcomes of all pregnancies in a remote northern community**. *CMAJ* 1991, **144(8)**:987–94.
45. Grzybowski S, Kornelsen J, Cooper E: **Rural maternity care services under stress: the experiences of providers.** *Can J Rural Med* 2007, **12(2)**:89–94.
46. Grzybowski S, Kornelsen J, Prinsloo L, Kilpatrick N, Wollard R: **Professional isolation in small rural surgical programs: the need for a virtual department of operative care**. *Can J Rural Med* 2011, **16(3)**:103–5.
47. Halm EA, Lee C, Chassin MR: **Is volume related to outcome in health care? A systematic review and methodological critique of the literature**. *Ann Intern Med*. 2002, **127**:511–20.
48. Hancock C, Steinbach A, Nesbitt TS, Adler SR, Auerswald CL: **Why doctors choose small towns: a developmental model of rural physician recruitment and retention**. *Soc Sci Med* **69(9)**:1368–76.

49. Hart LG, Amundson BA, Rosenblatt RA: **Is there a role for the small rural hospital?** *J Rural Health* 1990, **6(2)**:101–18.
50. Hart LG, Dobie SA, Baldwin LM, Pirani MJ, Fordyce M, Rosenblatt RA: **Rural and urban differences in physician resource use for low-risk obstetrics**. *Health Serv Res* 1996;31(4):429–52.
51. Hays RB, Evans RJ, Veitch C: **The determinants of quality in procedural rural medical care**. *Rural Remote Health* 2005a, **5(4)**:473.
52. Hays RB, Evans RJ, Veitch C: **The quality of procedural rural medical practice in Australia**. *Rural Remote Health* 2005b, **5(4)**:474.
53. Heneghan SJ, Bordley J, Dietz P a, Gold MS, Jenkins PL, Zuckerman RJ: **Comparison of urban and rural general surgeons: motivations for practice location, practice patterns, and education requirements**. *J Am Coll Surg* 2005 **201(5)**:732–6.
54. Hogenbirk JC, Mian O, Pong RW: **Postgraduate specialty training in northeastern Ontario and subsequent practice location**. *Rural Remote Health* 2011, **11(1)**:1603.
55. Hueston WJ: **Specialty differences in primary cesarean section rates in a rural hospital.** *Fam Pract Res J* 1992;**12(3)**:245–53.
56. Hueston WJ, Lewis-Stevenson S: **Provider distribution and variations in statewide cesarean section rates**. *J Community Health* 2001, **26(1)**:1–10.
57. Hueston WJ, Rudy M: **A comparison of labor and delivery management between nurse midwives and family physicians.** *J Fam Pract* 1993 **37(5)**:449.
58. Hulme PA, Blegen MA: **Residential status and birth outcomes: is the rural/urban distinction adequate?** *Public Health Nursing* 1999, **16(3)**:176–81.
59. Humber, N, Frecker, T: **Rural Surgery in British Columbia: Is There Anybody out There?** *Canadian Journal of Surgery*. 2008b, **51(3)**:179–84.
60. Humphreys JS, Jones MP, Jones J a, Mara PR: **Workforce retention in rural and remote Australia: determining the factors that influence length of practice.** *Med J Aust* 2002, **176(10)**:472–6.
61. Iglesias S, Burn R, Saunders LD: **Reducing the cesarean section rate in a rural community hospital**. *CMAJ* 1991, **145(11)**:1459–64.
62. Inglis FG: **The community general surgeon: a time for renaissance.** *Can J Surg*. 1995, **38(2)**: 123-129.
63. Jackson Pulver L, Haswell M, Ring I, Waldon J, Clark W, Whetung V, *et al*: *Indigenous Health – Australia, Canada, Aotearoa New Zealand and the US – Laying claim to a future that embraces health for us all*. WHO: World Health Organization; 2010.
64. Klein M, Johnston S, Christilaw J, Carty E: **Mothers, babies, and communities - Centralizing maternity care exposes mothers and babies to complications and endangers community stability**. *Can Fam Physician* 2002a **48**:1177–9.
65. Kornelsen JA, Grzybowski SW: **Obstetric services in small rural communities: what are the risks to care providers?** *Rural Remote Health* 2008, **8(2)**:943.
66. Kornelsen J, Grzybowski S: **Cultures of risk and their influence on birth in rural British Columbia**. *BMC Fam Pract* 2012, **13**:108.
67. Kornelsen J, Iglesias S, Humber N, Caron N, & Grzybowski S: **GP surgeons' experiences of training in British Columbia and Alberta: a case study of enhanced skills for rural primary care providers**. *Canadian Medical Education Journal* 2012, **3(1)**: 33-41.
68. Kornelsen J, Iglesias S, Humber N, Caron N, & Grzybowski S: **The Experience of GP Surgeons in Western Canada: The Influence of Interprofessional Relationships in Training and Practice**. *Journal of Research in Interprofessional Practice & Education* 2013, **3(1)**.
69. Kozhimannil KB, Law MR, Virnig BA: **Cesarean Delivery Rates Vary Tenfold Among US Hospitals; Reducing Variation May Address Quality and Cost Issues**. *Health Aff* 2013, **32(3)**:527–35.
70. Landercasper J, Bintz M, Cogbill TH, Bierman SL, Buan RR, Callaghan JP, *et al*: **Spectrum of general surgery in rural America**. *Arch Surg* 1997, **132(5)**:494–8.
71. Larimore WL, Davis A: **Relation of infant mortality to the availability of maternity care in rural Florida**. *J Am Board Fam Pract* 1991, **8(5)**:392–9.

72. Larson EH, Murowchick E, Hart LG: *Poor Birth Outcome in the Rural United States : 1985-1987 to 1995-1997*. The University of Washington Rural Health Resaerch Centre; 2008.
73. Laven, G, Wilkinson, D: **Rural Doctors and Rural Backgrounds: How Strong Is the Evidence? A Systematic Review**. Aust J Rural Health 2003, **11(6)**:277–84.
74. Leeman L, Leeman R: **A Native American community with a 7% cesarean delivery rate: does case mix, ethnicity, or labor management explain the low rate?** *Ann Fam Med* 2003, **1(1)**:36–43.
75. Leeman L, Leeman R: **Do all hospitals need cesarean delivery capability? An outcomes study of maternity care in a rural hospital without on-site cesarean capability.** *J Fam Pract* 2002, **51(2)**:129–34.
76. Lisonkova S, Sheps SB, Janssen PA, Lee SK, Dahlgren L, Macnab YC: **Birth outcomes among older mothers in rural versus urban areas: a residence-based approach**. *J Rural Health 2011*, **27(2)**:211–9.
77. MacDowell M, Glasser M, Fitts M, Fratzke M, Peters K: **Perspectives on rural health workforce issues: Illinois-Arkansas comparison**. *J Rural Health* 2009, **25(2)**:135–40.
78. MacDowell M, Glasser M, Fitts M, Nielsen K, Hunsaker M: **A national view of rural health workforce issues in the USA**. *Rural Remote Health* 2010, **10(3)**:1531.
79. MacLennan AH, Spencer MK: **Projections of Australian obstetricians ceasing practice and the reasons.** *Med J Aust* 2002, **176(9)**:425–8.
80. Magann EF, McKelvey SS, Hitt WC, Smith MV, Azam GA, Lowery CL: **The use of telemedicine in obstetrics: A review of the literature**. *Obstet Gynecol Surv* 2011, **66(3)**:170–8.
81. Mahoney SF, Malcoe LH: **Cesarean delivery in Native American women: are low rates explained by practices common to the Indian health service?** *Birth* 2005, **32(3)**:170–8.
82. Maouris P, Jennings B, Ford J, Karczub A, Kohan R, Butt J, et al: **Outreach obstetrics training in Western Australia improves neonatal outcome and decreases caesarean sections**. *J Obstet Gynaecol* 2010, **30(1)**:6–9.
83. McGrail MR, Humphreys JS: **The index of rural access: an innovative integrated approach for measuring primary care access**. *BMC Health Serv Res* 2009, **9**:124.
84. Miewald C, Procyk A: **"You don't know what you've got till it's gone": the role of maternity care in community sustainability**. *Can J Rural Med* 2011, **16(1)**:7–12.
85. Miller KJ, Couchie C, Ehman W, Graves L, Grzybowski S, Medves J: **Rural maternity care**. *J Obstet Gynaecol Canada* 2012, **34(10)**:984–91.
86. Mirabello J: **Getting to Havarti: Moving toward patient safety in obstetrics**. *Obstet Gynecol* 2008, **111(3)**:777.
87. Monk AR, Tracy S, Foureur M, Barclay L: **Australian primary maternity units: Past, present and future**. *Women and Birth* 2013, **26(3)**:213–8.
88. Mooney SE, Ogrinc G, Steadman W: **Improving emergency caesarean delivery response times at a rural community hospital**. *Qual Saf Heal Care* 2007, **16(1)**:60–6.
89. National Health Service Scotland: *Implementing a Framework for Maternity Services in Scotland*. http://www.scotland.gov.uk/Resource/Doc/47021/0013919.pdf
90. Nesbitt, TS, Connell, FA, Hart, FA, and Rosenblatt, RA: **Access to Obstetric Care in Rural Areas: Effect on Birth Outcomes** *American Journal of Public Health* 1999, **80(7)**:814–18.
91. Nesbitt, TS, Larson, EH, Rosenblatt, RA, and Hart, LG: **Access to Maternity Care in Rural Washington: Its Effect on Neonatal Outcomes and Resource Use.** *American journal of public health* 1997 **87(1)**:85–90.
92. New South Wales Ministry of Health: *Rural Surgical Futures 2011-2021* [Internet]. 2012.
93. Norris TE, Coombs JB, Carline J: **An educational needs assessment of rural family physicians.** *J Am Board Fam Pract* 1996, **9(2)**:86–93.
94. Norris TE, Reese JW, Pirani MJ, Rosenblatt RA: **Are rural family physicians comfortable performing cesarean sections?** *J Fam Pract 1996*, **43(5)**:455–60.
95. Northern Neonatal Network, Northern Regional Health Authority. (1993). Requirements for neonatal cots. Archives of Disease in Childhood 68: 544-549.
96. Odibo IN, Wendel PJ, Magann EF: **Telemedicine in obstetrics.** *Clin Obstet Gynecol* 2013, **56(3)**:422–33.
97. Office of Rural Health and Primary Care- Minnesota Dept of Health. *Rural Health Advisory Committee Report on Obstetric Services in Rural Minnesota*. 2013. http://www.health.state.mn.us/divs/orhpc/rhac/obrpt.pdf

98. Oxenham J: **What it's like to work in... Shropshire**. *Pract Midwife* 2002 **5(9)**:27–9.
99. Pong RW, Pitblado JR: *Geographic distribution of physicians in Canada: beyond how many and where*. Ottawa: Canadian Institute for Health Research (CIHI); 2005.
100. Powell J, Dugdale AE: **Obstetric outcomes in an aboriginal community: a comparison with the surrounding rural area**. *Aust J Rural Health* 1999, **7(1)**:13–7.
101. Power R: **General practitioner obstetric practice in rural and remote Western Australia**. *Australian and New Zealand journal of obstetrics and gynaecology* 1995, **35(3)**: 241-244.
102. Powers JR, Loxton DJ, O'Mara AT, Chojenta CL, Ebert L: **Regardless of where they give birth, women living in non-metropolitan areas are less likely to have an epidural than their metropolitan counterparts**. *Women and Birth* 2013, **26(2)**:77–81.
103. Prior M, Farmer J, Godden DJ, Taylor J: **More than health: the added value of health services in remote Scotland and Australia**. *Health Place* 2010, **16(6)**:1136–44.
104. Queensland Government: *Clinical Services Capability Framework for Public and Licensed Private Health Facilities version 3.1 - Maternity Services Module*. 2012. http://www.health.qld.gov.au/cscf/
105. Rabinowitz HK, Diamond JJ, Markham FW, Wortman JR: **Medical school programs to increase the rural physician supply: a systematic review and projected impact of widespread replication**. *Acad Med* 2008, **83(3)**:235–43.
106. Renwick MY: **Caesarean section rates, Australia 1986: variations at state and small area level**. *Aust N Z J Obstet Gynaecol* 1991, **31(4)**:299–304.
107. Roberts CL, Algert CS, Peat B, Henderson-Smart D: **Differences and trends in obstetric interventions at term among urban and rural women in New South Wales: 1990-1997**. *Aust New Zeal J Obstet Gynaecol* 2001, **41(1)**:15–22.
108. Rodney WM, Martinez C, Collins M, Laurence G, Pean C, Stallings J: **OB fellowship outcomes 1992-2010: where do they go, who stops delivering, and why?** *Fam Med* 2010, **42(10)**:712–6.
109. Rosenthal TC: **Outcomes of rural training tracks: A review**. *J Rural Health* 2000, **16(3)**:213–6.
110. Rosenthal DR: *The achievement and maintenance of emergency medicine standards in rural practice*. 2001. http://www.hinz.org.nz/journal/2001/06/The-Achievement-and-Maintenance-of-Emergency-Medicine-Standards-in-Rural-Practice/543
111. Rosenthal TC, Ferrara E, Hesler E: **Providing birthing services in rural health networks: coping with change in New York State**. *J Rural Health* 1996, **12(2)**:137–45.
112. Rosenthal TC, McGuigan MH, Osborne J, Holden DM, Parsons MA: **One-two rural residency tracks in family practice: are they getting the job done?** *Fam Med* 1998, **30(2)**:90–3.
113. Rourke J, Frank JR: **Implementing the CanMEDSTM physician roles in rural specialist education: the Multi-Speciality Community Training Network**. *Rural Remote Health* 2005, **5(4)**:406.
114. Royal Australasian College of Surgeons: *Training for GP Surgical Proceduralists*. 2010. http://www.surgeons.org/media/8524/FES_RSE_2360_P_Position_Paper_Training_for_GP_Surgical_Proceduralists.pdf
115. Sariego J: **Patterns of surgical practice in a small rural hospital**. *J Am Coll Surg* 1999, **189(1)**:8–10.
116. Schauer RW, Schieve D: **Performance of medical students in a nontraditional rural clinical program, 1998-99 through 2003-04**. *Acad Med* 2006, **81(7)**:603–7.
117. Schultz R, Lockey R, Oats JJ: **Birthing in the Barkly: births to Barkly women in 2010.** *Rural Remote Health* 2013, **13(3)**:2396.
118. Serenius F, Winbo I, Dahiquist G, Källén B: **Cause-specific stillbirth and neonatal death in Sweden: a catchment area-based analysis**. *Acta Paediatr* 2001, **90(9)**:1054–61.
119. Shively EH, Shively S: **Threats to rural surgery**. *Am J Surg* 2005, **190(2)**:200–5.
120. Smith et al. (2009) **Ontario Care Providers' Considerations Regarding Models of Maternity Care.** Journal of Obstetrics and Gynaecology Canada. 31(5):401-408
121. Society of Obstetricians and Gynaecologists of Canada [SOGC]: *A national birthing initiative for Canada: An inclusive, integrated and comprehensive pan-Canadian framework for sustainable family-centered maternity and newborn care*. 2008.

122. Sticca RP, Mullin BC, Harris JD, Hosford CC: **Surgical specialty procedures in rural surgery practices: Implications for rural surgery training**. *Am J Surg* 2012, **204(6)**:1007–13.
123. Stratigos S, Nichols A: *Procedural Rural Medicine: Strategies towards solutions: A paper prepared for the AHMAC National Rural Health Policy*. 2002. http://www.rdaa.com.au/Uploads/Documents/PROCEDURAL%20RURAL%20MEDICINE%20strategies%20towards%20solutions%20FINAL.%202%20pdf_20101012034305.pdf
124. Swindlehurst HF, Deaville JA, Wynn-Jones J, Mitchenson K: **Rural Proofing for Health: A commentary**. *Rural Remote Heal*. 2005, **5(411)**.
125. The Ontario Joint Policy and Planning Committee: *The Core Service Role of Small Hospitals in Ontario*. 2006. http://ruralontarioinstitute.ca/file.aspx?id=06f58a3b-ee30-46eb-84c2-e5ee1b718733.
126. The Ontario Rural Council: *The TORC Report on Rural Health: Rethinking Rural Health Care:Innovations Making a Difference*. 2009. http://ruralontarioinstitute.ca/file.aspx?id=1fb3035d-7c0e-4bfa-a8d7-783891f5c5dc
127. Thommasen H V, Klein MC, Mackenzie T, Lynch N, Reyes R, Grzybowski S: **Obstetric maternal outcomes at Bella Coola General Hospital: 1940 to 2001**. *Canadian Journal of Rural Medicine* 2005, **10(1)**:13–21.
128. Thompson MJ, Lynge DC, Larson EH, Tachawachira P, Hart LG: **Characterizing the general surgery workforce in rural America**. *Archives of Surgery* 2005, **140(1)** :74–9.
129. Tucker J, du V. Florey C, Howie P, McIlwaine G, Hall M: **Is antenatal care apportioned according to obstetric risk? The Scottish antenatal care study**. *J Public Health Med* 1994, **16(1)**:60–70.
130. Tulloh B, Clifforth S, Miller I: **Caseload in rural general surgical practice and implications for training**. *ANZ J Surg* 2001, **71(4)**:215–7.
131. VanBibber M, Zuckerman RS, Finlayson SRG: **Rural versus urban inpatient case-mix differences in the US**. *Journal of the American College of Surgeons* 2006, **203(6)**: 812–6.
132. Wakerman J, Humphreys JS, Wells R, Kuipers P, Entwistle P, Jones J: **Primary health care delivery models in rural and remote Australia: a systematic review**. *BMC Health Serv Res* 2008, **8(1)**:276.
133. Watts RW, Marley JE, Beilby JJ, MacKinnon RP, Doughty S: **Training, skills and approach to high-risk obstetrics in rural GP obstetricians**. *Aust New Zeal J Obstet Gynaecol* 1997, **37(4)**:424–6.
134. Williams Jr. TE, Satiani B, Ellison EC: **A comparison of future recruitment needs in urban and rural hospitals: The rural imperative**. *Surgery* 2011: 150(4):617–25.
135. Worley P: Flinders University School of Medicine, Northern Territory, Australia: **Achieving Educational Excellence along with a Sustainable Rural Medical Workforce**. *MEDICC Rev* 2008;10(4):30–4.
136. Zelek B, Orrantia E, Poole H, Strike J. **Home or away? Factors affecting where women choose to give birth**. *Can Fam Physician* 2007, **53(1)**:78–9.
137. Zuckerman R, Doty B, Gold M, Bordley J, Dietz P, Jenkins P, et al: **General surgery programs in small rural New York State hospitals: a pilot survey of hospital administrators**. *J Rural Health* 2006, **22(4)**:339–42.
138. Zust BL, Briggs NB: **Labor induction practices in a rural midwestern hospital**. *Online J Rural Nurs Heal Care* 2006, **6(2)**.
